# Transport Properties of Mie Fluids: Molecular Dynamics Simulations and Entropy Scaling

**DOI:** 10.1021/acs.jpcb.6c04595

**Published:** 2026-10-15

**Authors:** Dennis Alt, Simon Stephan

**Affiliations:** † Molecular Thermodynamics Group (MTG), 249761 RPTU Kaiserslautern, Kaiserslautern 67663, Germany; ‡ Laboratory of Heat and Mass Transfer (WSÜ), 9376 OVGU Magdeburg, Magdeburg 39106, Germany

## Abstract

Transport properties of pure Mie fluids and Mie fluid mixtures were studied using molecular dynamics (MD) simulations and fluid theory (FT). The molecular simulation results are rigorously compared with fluid theory model results, i.e., entropy scaling (ES) in combination with a molecular-based equation of state (EOS). The agreement of the results from MD and ES + EOS is good overall. The following transport coefficients were studied: shear viscosity, thermal conductivity, self-diffusion coefficients, and mutual diffusion coefficients, including both Fickian and *Maxwell–Stefan* diffusion coefficients. The focus was on the variation of the repulsive Mie potential exponent (*n* = 8, 10, 12, 14, 16, 20, 36, 48 with the dispersive exponent constant at *m* = 6). Also, the influence of the dispersive Mie potential exponent was studied (*m* = 4, 5, 6, 7, 8 with the repulsive exponent constant at *n* = 12). Additionally, Mie fluids with (*n*, *m*) ∈ [(20, 8); (20, 4); (8, 4)] were studied. In total, 15 pure Mie fluids—for each more than 200 state points—were studied by MD simulations covering liquid, gaseous, and supercritical phases. Moreover, two binary mixtures with (*n*, *m*) ∈ [(12, 5); (16, 6)] and (*n*, *m*) ∈ [(14, 5); (20, 7)] exponents were investigated, focusing on the mixture diffusion coefficients. Therefore, the ES diffusion framework from SchmittS.,  [Nat. Commun. 2025, 16, 2611]10.1038/s41467-025-57780-zPMC1191449240097384 was deployed. The predictions from the FT framework are in good overall agreement with the molecular simulation reference data, with the best agreement for self-diffusion coefficients. In all cases, a wide range of thermodynamic conditions, i.e., temperature, pressure, and composition, was investigated.

## Graphical abstract

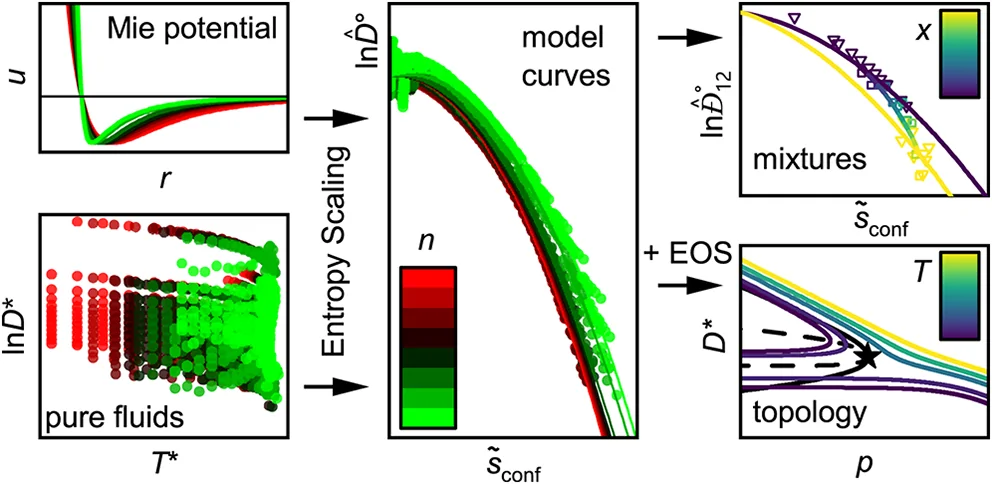

## Introduction

The accurate modeling of transport properties across a wide range of thermodynamic conditions is important for many applications, e.g., tribology,^1,2^ chemical engineering,^3–6^ and astronomy.^7,8^ Since experimental data for transport coefficients such as the shear viscosity, thermal conductivity, and diffusion coefficients are scarce, models with strong predictive capabilities are required. Fluid-theory models from molecular thermodynamics provide an attractive tool for modeling thermophysical properties.^9–15^ Due to their physical backbone, fluid-theory models enable predictions far beyond the range where experimental data are available. For the validation and refinement of the physical basis of fluid-theory frameworks, model fluids such as the Mie fluid can be favorably used. Such model fluids can be evaluated directly via molecular simulation to obtain reference data for validating fluid-theory predictions and identifying specific shortcomings in the theory. The Mie (*n*,*m*) fluid provides a particularly suitable model for such an analysis because the repulsive and attractive interactions can be adjusted independently, enabling a controlled investigation of how the shape of the interaction potential affects the agreement of fluid theory and molecular simulation reference data.^44^ Additionally, this provides insight into the influence of the potential exponents on transport properties.

The Mie fluid is the generalized form of the *Lennard-Jones* (LJ) fluid.^16–18^ The Mie fluid has been extensively used for elucidating the influence of the shape of the intermolecular potential on macroscopic properties, e.g., vapor–liquid equilibria,^19–23^ virial coefficients,^24–26^ and Brown’s characteristic curves.^27,28^ In the Mie potential, the classical *n* = 12, *m* = 6 exponents of the LJ potential^16^ are variable parameters. In the LJ potential, the choice for the dispersive exponent *m* = 6 is physically motivated,^29^ whereas the repulsive exponent *n* = 12 was originally primarily chosen for computational convenience.^30^

While transport properties of the *Lennard-Jones* fluid have been extensively studied in the literature,^31–33^ corresponding data and transferable models for Mie fluids remain comparatively limited. Eskandari Nasrabad et al.^34^ reported *Green–Kubo* MD data for the self-diffusion coefficient, shear viscosity, and thermal conductivity of the Mie (14, 7) fluid and compared selected results to theoretical models. Delage-Santacreu et al.^35^ used nonequilibrium MD to investigate thermodynamic scaling of the shear viscosity of Mie (*n*, 6) fluids with *n* = 8, 12, 18, 24, 36 and selected binary mixtures. More recently, Šlepavičius et al.^36^ generated MD data for self-diffusion and shear viscosity for 54 Mie potentials and correlated the data using empirical models. Also, Chaparro and Müller^37^ reported MD data for Mie fluids for the self-diffusion coefficient, shear viscosity, and thermal conductivity of Mie (*n*, 6) fluids and proposed empirical correlations of the data. Saric et al.^38^ reported shear viscosity, self-diffusion, and thermal conductivity data for four Mie (*n*, 6) fluids with *n* = 9, 12, 15, 18 (among other monatomic potentials) and analyzed the scaling behavior of that data. Thus, transport properties of pure component Mie fluids have at times been studied in the literature before – especially by MD simulations – but mainly for Mie (*n*, 6) fluids or individual exponent pairs. No mutual diffusion coefficient data of Mie fluids are available in the literature – to the best of our knowledge. Studies focusing on fluid-theory modeling of Mie fluid transport properties are even scarcer,^35,39^ particularly for mixtures. Moreover, most studies focus on the variation of the repulsive exponent solely. A comparative discussion on possible routes for modeling transport properties of Mie fluids is given in the Supporting Information.

Few physically motivated fluid-theory frameworks are available for modeling transport properties, e.g., entropy scaling^40,41,45–49^ and friction theory.^50^ Both are add-on models that couple static equilibrium properties (i.e., entropy or pressure) described by a fundamental equation of state (EOS) with transport coefficients (e.g., the viscosity and diffusion coefficients). In this work, entropy scaling (ES) was used. ES is based on the finding that reduced transport coefficients of fluids collapse approximately onto monovariate functions with respect to the configurational entropy, i.e., the entropy contribution arising from the intermolecular interactions. In the literature, this quantity is also referred to as residual or excess entropy. Throughout this work, the term configurational entropy is used. For simple fluids, this behavior is commonly rationalized using isomorph theory, which links reduced structural and dynamical invariance to contours of constant configurational entropy.^47^ Within the entropy scaling framework, the configurational entropy has moreover been used to define a demarcation between gas- and liquid-like dynamics. This is defined by the point at which the individual and the collective contributions to the viscosity coincide.^42^ Several executable ES frameworks have been proposed in the past years in the literature, e.g., refs 10,33,41,48,51 . In this work, we use the entropy scaling framework from a previous work of our group,^48^ which was specifically designed to be coupled with molecular-based EOS. The ES framework from Schmitt et al.^48^ is designed for modeling the shear viscosity, thermal conductivity, and self-diffusion coefficient of pure fluids as well as the shear viscosity and thermal conductivity of fluid mixtures, where additional assumptions are required, e.g., regarding the effective molecular mass.^43^ It has been successfully deployed for modeling both model fluids and real substances.^52–54^ For modeling diffusion coefficients in fluid mixtures, a novel approach was developed in our group^6^ that enables the simultaneous and consistent modeling of self-diffusion and mutual diffusion coefficients in binary mixtures. This novel framework has not yet been comprehensively tested. In this work, we use Mie fluids and Mie fluid mixtures for rigorously validating the ES framework proposed in refs 6 and 48.

The SAFT-VR Mie EOS^9^ was used in conjunction with the ES framework. In previous works of our group, we have evaluated different molecular-based EOS for modeling various thermodynamic properties^55–58^ and found that the SAFT-VR Mie EOS yields in practically all cases the most accurate and robust description, which is consistent with findings from other groups.^59–62^ Therefore, the SAFT-VR Mie EOS was deployed in this work for modeling static thermodynamic properties of the Mie fluids.

The Mie fluid model class is a particularly interesting candidate for investigating the fundamentals of ES. Because the repulsive part of Mie interactions can often be approximated by an effective inverse-power-law, varying (*n*,*m*) is expected to primarily shift scaling prefactors and density dependence while preserving, to first order, the shape of the reduced entropy scaling master curve.^40,63^ Consequently, similar local slopes are expected when the reduced transport properties are plotted against the same entropy variable. However, exact equality is not expected, and systematic deviations may arise at low density, near criticality, or in structurally anomalous/associating regimes.^47^ This is consistent with the findings of Saric et al.,^38^ who reported that the repulsive steepness, controlled by *n*, has only a minor influence on the quality of entropy scaling for the Mie (*n*, 6) family within the range they considered. In that study,^38^ the scaling behavior of four Mie (*n*, 6) fluids was evaluated based on MD data, but no transferable, executable fluid-theory model (e.g., an EOS-coupled predictive framework) was developed and applied. Moreover, by studying both pure components and mixtures, we can assess a possible accuracy decay of the fluid theory going from pure components to mixtures, which goes hand in hand with additional assumptions and approximations, e.g., regarding the one-fluid picture in the EOS and isomorph theory in the ES kernel.

In this work, a complementary study of computer experiments and fluid theory was conducted: MD simulations were carried out for generating reference data for transport coefficients for various pure component and mixture Mie fluids in a wide range of thermodynamic states (temperature, pressure, composition). This comprehensive data set was used to rigorously validate a recently developed entropy scaling framework.^6,48^ This paper is structured as follows: First, the employed methods are described; then, the results from different methods are compared and discussed. The numerical data are provided in the Supporting Information. All calculations were carried out in “*Lennard-Jones*” reduced units, i.e., the particle molar mass *M*, segment number *m*_s_, segment diameter σ, and segment dispersion energy ε were set to unity. All results are reported using this unit system.

## Methods

### Studied Mie Fluids

In this work, both pure component Mie fluids as well as binary mixtures of Mie fluids were studied. Mie fluids are defined as a substance class where the particles interact according to the Mie interaction potential. The Mie potential *u*_Mie_ ^64^ is mathematically defined as

$$u_{\mathrm{Mie}}\left(r\right)=\frac{n}{n-m}{\left(\frac{n}{m}\right)}^{\frac{m}{n-m}}\varepsilon \left[{\left(\frac{\sigma }{r}\right)}^{n}-{\left(\frac{\sigma }{r}\right)}^{m}\right]$$ 1

where *n* and *m* denote the repulsive and attractive (dispersive) exponents, respectively, σ and ε are the size and energy parameters, and *r* is the intermolecular distance between two particles. The repulsive exponent *n* mainly influences the slope of the Pauli repulsion for *r* → 0, hence increasing *n* yields harder repulsion. The dispersive exponent *m* primarily determines the strength of the attractive forces in the potential. Despite the fact that repulsion and dispersion are described by two separate terms in the Mie potential, both terms in eq 1 influence the potential form in the entire range of *r*, cf. Figure 1: The variation of the repulsive exponent *n* also influences the form of the potential in the attractive part and the dispersive exponent *m* also influences the repulsive part of the potential. Moreover, the dispersive exponent “6” is a result of approximations and assumptions in *London*’*s* derivation for dispersion^65^ – despite the fact that it is often referred to as “being a physical fact”. Thus, the dispersive exponent 6 should not be regarded as immutable, and its influence on thermophysical properties is of general interest.

**Figure 1:**
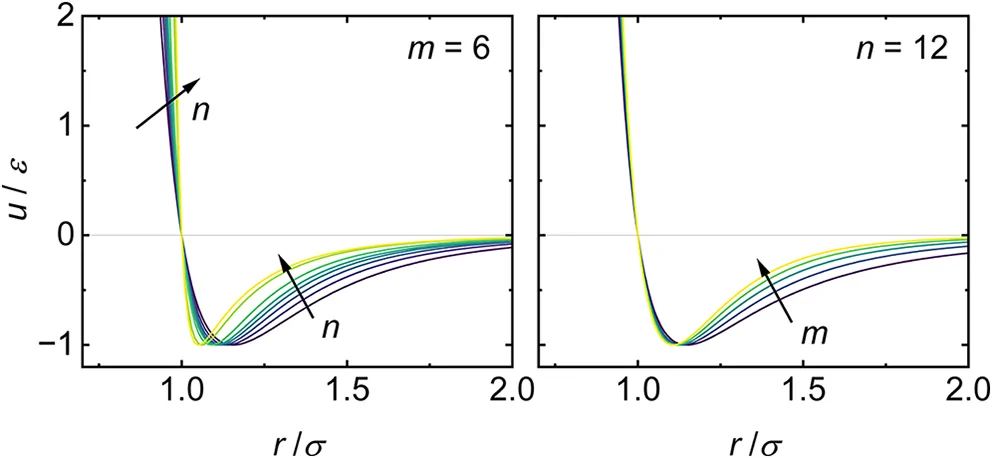
Influence of the parameters *n* and *m* on the shape of the Mie potential. Left: *m* = 6, *n* ∈ {8, 10, 12, 14, 16, 20, 36, 48}; Right: *n* = 12, *m* ∈ {4, 5, 6, 7, 8}.

In this work, 15 pure Mie (*n*, *m*) substances and two binary mixtures were investigated (see Figure 2). For the pure substances, both members of the orthogonal exponent families with fixed repulsive or attractive exponent (*n* = 12 and *m* = 6, respectively) were considered, together with additional mixed-exponent cases (Mie (20, 8); Mie (20, 4); and Mie (8, 4)). Binary mixture A consists of the previously characterized substances Mie (12, 5) and Mie (16, 6), whereas binary mixture B comprises Mie (14, 5) and Mie (20, 7). This selection enables a systematic separation of repulsive and attractive exponent effects and also enables testing the transferability and predictive capabilities of the fluid theory for mixed-exponent fluids and binary mixtures. In all cases, the size parameter σ and energy parameter ε were unity.

**Figure 2:**
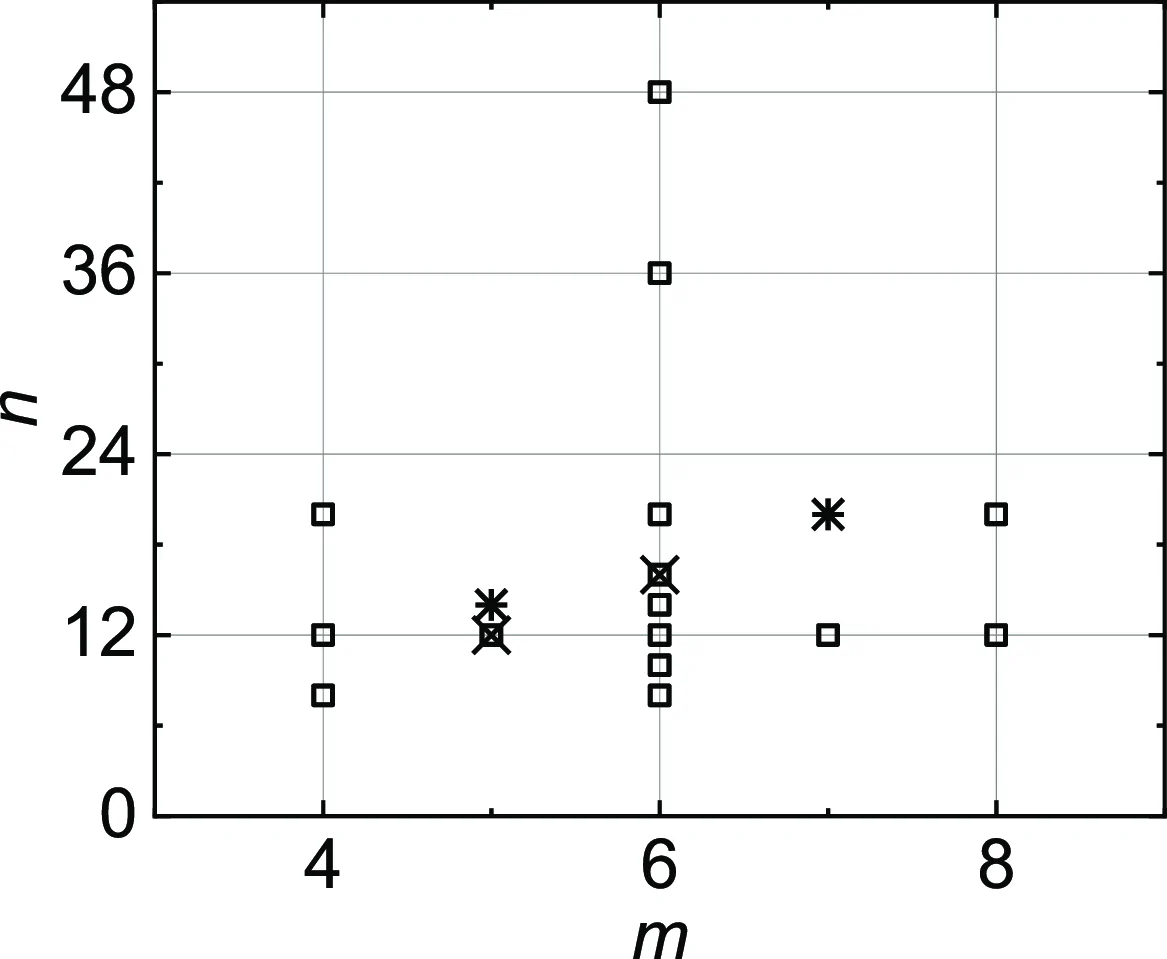
Mie (*n*,*m*) substances and mixtures studied in this work. Squares: Pure substances; Crosses: Binary mixture A; Asterisks: Binary mixture B.

### Studied Thermodynamic States

Both the pure components and binary mixtures were studied in a wide range of states using MD simulations. For each pure component, at least 206 states were studied, for each binary mixture, at least 115 states were studied. The state points for the pure fluid simulations were chosen using critical reduced units, i.e., in terms of *T*/*T*_c_ and ρ/ρ_c_ using the critical temperature and density of a given Mie fluid. Thereby, gas, liquid, supercritical, and metastable states were covered in a comparable manner across different Mie fluids (cf. Figure 3).

**Figure 3:**
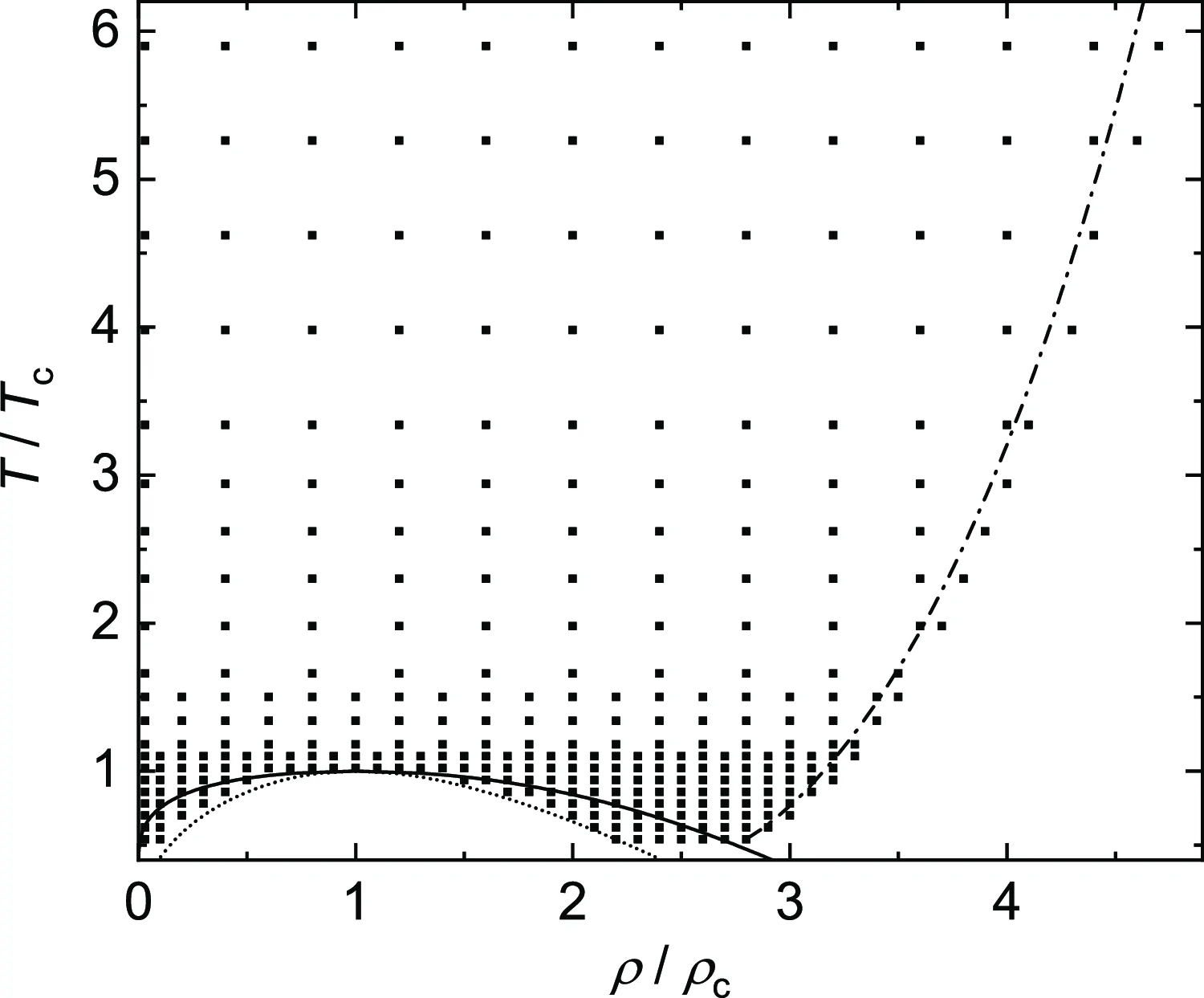
Simulation grid for pure substances in critical reduced units. Black solid squares indicate the state points studied in this work. The dash-dotted line shows the melting line from Schultz and Kofke^66^ (for the *Lennard-Jones* substance). The binodal (solid line) and spinodal (dotted line) of the VLE were calculated by the equation of state from Kolafa and Nezbeda^67^ for the *Lennard-Jones* substance.

For Mie fluids with *n* = 12 or *m* = 6, the critical temperature *T*_c_ and critical density ρ_c_ were taken from the correlations of Stephan and Urschel.^27^ For mixed-exponent Mie fluids with *n* ≠ 12 and *m* ≠ 6 (i.e., Mie exponents (8, 4); (20, 4); and (20, 8)), the critical properties were determined in this work. Therefore, dedicated vapor–liquid equilibrium molecular simulations were carried out. Those were carried out with the code *ms*2 ^68,69^ using the grand equilibrium (GE) method of *Vrabec and Hasse*,^70^ initialized with vapor–liquid equilibrium (VLE) estimates from the SAFT-VR Mie equation of state (EOS).^9^ To refine the coexistence data, three consecutive GE + *NpT* simulation cycles were performed, after which the critical point was estimated from the resulting VLE data. Details are given in the Supporting Information, and the primary VLE data are provided in the Supporting Information. The resulting critical properties are reported in Table 1.

**Table 1:** Critical Data for *n* ≠ 12 and *m* ≠ 6 Mie Fluids Obtained from GE Simulations Carried Out in This Work

*n*	*m*	*T*_c_^*^	ρ_c_^*^	*p*_c_^*^
8	4	4.8240	0.34711	0.48688
20	4	2.3929	0.30264	0.22618
20	8	0.77038	0.35275	0.078517

For the binary mixtures, the selection of state points was adopted from a previous work of our group^71^ (cf. Figure 4). For the mixtures, the focus was on liquid and supercritical states, where unlike interactions play a dominant role, resulting in nonideal behavior of the macroscopic properties. This challenges the entropy scaling framework. For each mixture, five isotherms were studied; for each isotherm, at least two pressure values were studied; for one temperature, four pressure values were studied. For each temperature–pressure pair, 11 composition values were studied. The compositions were sampled on the grid *x*_*i*_ ∈ {0.001, 0.1, 0.2, ..., 0.9, 0.999} mol·mol^–1^, thereby approximating the pseudo-pure limits^6^ while avoiding the singular end points *x*_*i*_ = 0 and 1 mol·mol^–1^.

**Figure 4:**
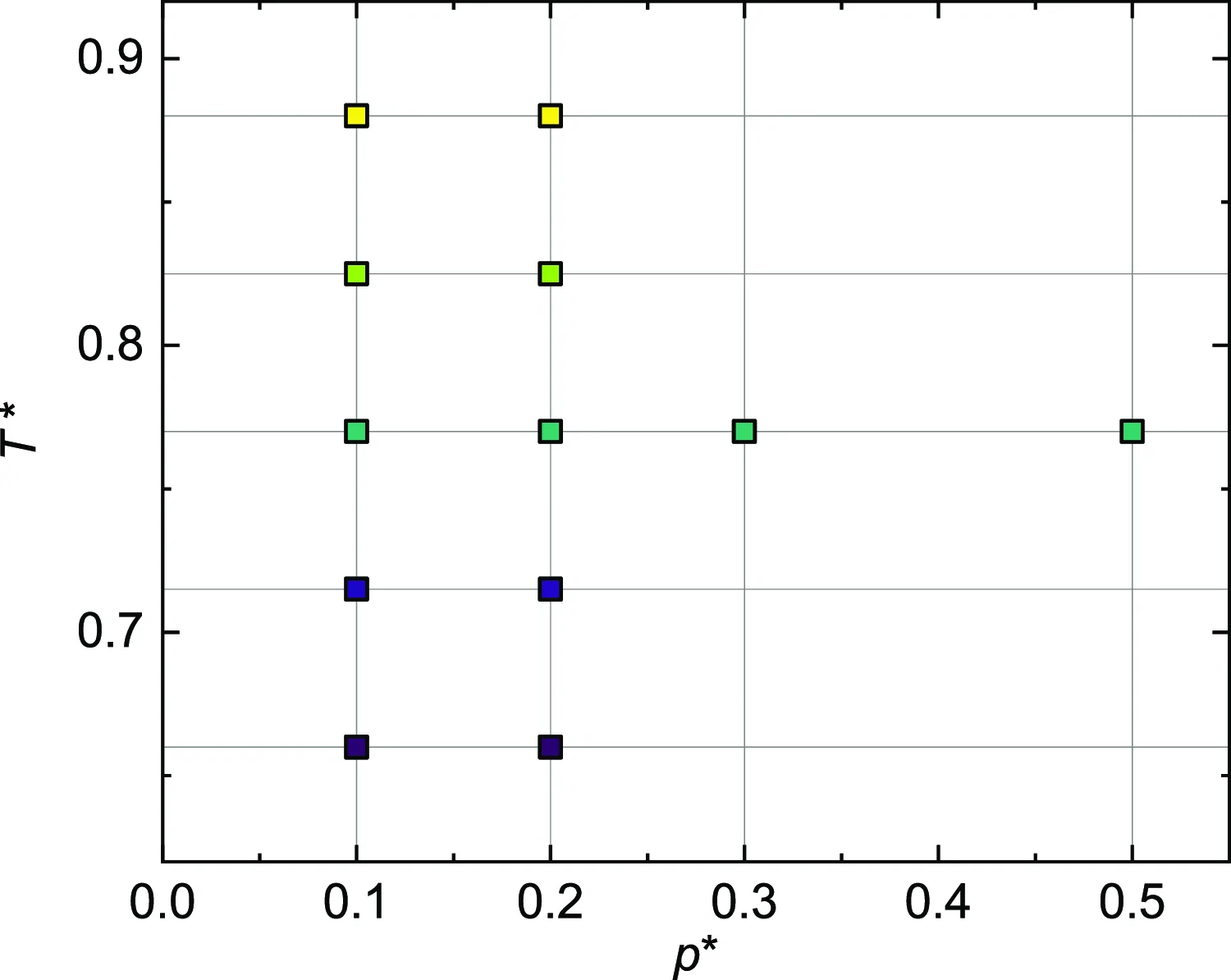
Simulation grid for mixtures in classical *Lennard-Jones* reduced units. Squares indicate the state points studied in this work. The colors are used throughout this work and indicate the temperatures T* ∈ {0.66, 0.715, 0.77, 0.825, 0.88}.

### MD Simulations

Molecular dynamics (MD) simulations were performed for sampling transport properties using the software package *ms*2.^68,69^ The equations of motion were integrated using a gear predictor-corrector algorithm. All pure substance simulations were conducted in the canonical ensemble (*NVT*), with constant temperature, density, and composition. Each system consisted of *N* = 4096 particles in a cubic simulation box with periodic boundary conditions applied in all directions. For all simulations, the Mie potential was used with a cutoff at 5 σ in combination with long-range corrections for pressure and internal energy. The dispersive tail of the different Mie potentials varies such that this cutoff for explicitly considering interactions might have somewhat different consequences for different Mie fluids. Nevertheless, for all Mie fluids, long-range corrections were applied.

Equilibration was performed over 800,000 time steps, followed by a production run of 4,000,000 time steps. Temperature control was ensured via velocity scaling.^72^ Thermostatting can, in principle, perturb dynamical correlations when its coupling acts on time scales comparable to the correlation decay.^73^ However, velocity scaling constrains only a single global quantity, the total kinetic energy, by rescaling all particle velocities with a common factor. Consequently, it introduces no stochastic forces or modifications of the interparticle forces. Since the temperature fluctuates by the order of *N* ^–0.5^ around its set value, the rescaling factor deviates only marginally from unity for the system size used in this work. Hence, the perturbation accumulated over the decay time of the *Green–Kubo* integrals is small in relation to their statistical uncertainty. Statistical uncertainties were estimated using the block averaging method proposed by Flyvbjerg and Petersen,^74^ in combination with the standard law of error propagation. The transport properties were sampled using the *Green–Kubo* formalism.^75,76^ The sampling parameters for the *Green–Kubo* autocorrelation functions were specified with respect to temperature and density to ensure good statistics in the sampling. The empirical model for the freezing-line ρ_s_(*T*), reported by Schultz and Kofke,^66^ was used to define the autocorrelation-function sampling parameters as well as the time step Δτ* (cf. Table 2). Although this correlation was originally derived for the *Lennard-Jones* fluid, it was adapted here for Mie fluids via corresponding states principle based on the critical properties.

**Table 2:** Simulation Parameters for MD Pure Component Simulations as a Function of the Density Relative to the Calculated Freezing-Line Density, *ρ*_s_(*T*)­^a^

density range	parameter	value
ρ ≤ 0.1 ρ_s_	*N*_step_	20
	*N*_span_	4000
	*N*_length_	200,000
0.1 ρ_s_ < ρ ≤ 0.2 ρ_s_	*N*_step_	10
	*N*_span_	1000
	*N*_length_	50,000
0.2 ρ_s_ < ρ	*N*_step_	5
	*N*_span_	200
	*N*_length_	10,000
ρ ≤ 0.01 ρ_s_	Δτ*	0.010
0.01ρ_s_ < ρ ≤ 0.05ρ_s_	Δτ*	0.005
0.05ρ_s_ < ρ	Δτ*	0.001

a Here, *N*_step_ denotes the sampling interval (i.e., how often correlation functions are evaluated), *N*_span_ is the number of time steps between the starts of successive correlation functions, *N*_length_ is the number of time steps used in the averaging of each correlation function, and Δτ* is the time step in reduced units (with respect to σ and ε).

For the mixture simulations, an initial simulation was performed in the isobaric–isothermal ensemble (*NpT*) to determine the equilibrium density at the specified temperature, pressure, and composition. Each simulation was conducted using *N* = 4096 particles and comprised 1,500,000 time steps for both the equilibration and the production phases with a time step of Δτ* = 0.005. The pressure was controlled using *Andersen*’*s* barostat.^72^ The densities ρ* obtained from the *NpT* simulations were subsequently used as input for a second simulation in the canonical ensemble (*NVT*), in which the transport properties were sampled. The mixture simulations in the *NVT* ensemble were performed using the parameter settings given in Table 3.

**Table 3:** Simulation Parameters for MD Mixture Simulations^a^

parameter	value
*N*_step_	5
*N*_span_	200
*N*_length_	10,000
Δτ*	0.001

a Here, *N*_step_ denotes the sampling interval (i.e., how often correlation functions are evaluated), *N*_span_ is the number of time steps between the starts of successive correlation functions, *N*_length_ is the number of time steps used in the averaging of each correlation function, and Δτ* is the time step in reduced units.

In the pure component simulations, three transport properties – the shear viscosity, the thermal conductivity, and the self-diffusion coefficient – were directly sampled in the simulations in the *NVT* ensemble. For that, the *Green–Kubo* formalism^75,76^ was employed. In the case of the binary mixtures, two self-diffusion coefficients as well as the *Maxwell–Stefan* mutual diffusion coefficient were sampled. Transport coefficients sampled from finite systems are subject to system-size effects. For the self-diffusion coefficient, the leading hydrodynamic contribution is given by the correction proposed by Yeh and Hummer.^77^ A corresponding correction for *Maxwell-Stefan* diffusion coefficients, which additionally accounts for the nonideality of the mixture, was proposed by Jamali et al.^78,79^ For the system size used here (*N* = 4096), these corrections amount to 6% and 8% (median over all state points), with maxima of 14% and 23% in the densest liquid states for self- and mutual diffusion, respectively. Here, we report the primary data sampled in the simulations. The quantities required for the evaluation of the corrections – the shear viscosity and the thermodynamic factor – are provided for every state point in the Supporting Information.

To determine the configurational entropy from molecular simulations, the configurational chemical potential was sampled. The expression

2 $$\mu_{\mathrm{conf}}=T\left({ã}_{\mathrm{conf}}+\rho \frac{d{ã}_{\mathrm{conf}}}{d\rho }\right)$$

enables the computation of the reduced configurational Helmholtz energy *ã*_conf_ = *a*_conf_/*T*. The configurational entropy *s*_conf_ can then be calculated as

3 $$s_{\mathrm{conf}}=\frac{1}{T}\frac{d{ã}_{\mathrm{conf}}}{d\left(1/T\right)}-{ã}_{\mathrm{conf}}$$

For the sampling of the chemical potential, two different methods – depending on the density of the state points – were employed in this work. For low densities (approximately ρ ≤ 0.9 ρ_s_ with ρ_s_(*T*) being the freezing density), the Widom particle insertion method^80^ was applied, using 4096 test particles. It samples the configurational chemical potential by randomly inserting virtual test particles into the system and averaging the Boltzmann factor of the resulting energy change. However, at high densities, the probability of overlap between test particles and existing particles increases, leading to poor statistical sampling. As a result, the Widom method becomes unsuitable for high-density liquid states. For cases, where the Widom method approaches its limitations, the chemical potential was determined using the thermodynamic integration method.^81^ Thermodynamic integration calculates the difference in chemical potential between two states by gradually introducing a particle into the system using a coupling parameter, which scales the interaction energy. The chemical potential difference is then obtained by integrating the ensemble average of the derivative of the potential energy with respect to the coupling parameter. The sampling parameters for the thermodynamic integration method used in this work are given in the Supporting Information.

For the sake of completeness, we have computed the Fickian diffusion coefficients *D*_*ij*_ using the sampled *Maxwell–Stefan* diffusion coefficient results *Đ*_*ij*_ and the thermodynamic factor Γ_*ij*_ using the relation

4 $$D_{\mathrm{ij}}=\Gamma_{\mathrm{ij}}{Đ}_{\mathrm{ij}}$$

The thermodynamic factor was computed using the SAFT-VR Mie EOS.^9^ The results for the Fickian diffusion coefficients are reported in the Supporting Information.

The computer experiment primary data were screened using thermodynamic and numerical consistency criteria. State points were excluded if at least one of the following conditions was met: (i) nucleation and phase split at metastable states – in particular freezing, (ii) nonconvergent or numerically unstable chemical-potential sampling (Widom insertion or thermodynamic integration), or (iii) thermodynamic inconsistencies in Helmholtz-energy derivatives indicating unreliable state point characterization (regarding *pvT*). Only individual data points had to be discarded using these consistency tests while preserving the overall continuity of the sampled state-space coverage.

### Fluid Theory

#### Entropy Scaling

Transport property modeling was performed using the entropy scaling framework developed by Schmitt et al.^48^ The core of the framework is a modified Rosenfeld scaling,^45,48,82^ which expresses dimensionless, reduced transport properties as functions of the configurational entropy:

5 $$\eta^{o}=\eta^{*}\frac{\rho_{N}^{*-2/3}}{\sqrt{T^{*}}}{\left(-s_{\mathrm{conf}}^{*}\right)}^{2/3}$$

6 $$\lambda^{o}=\lambda^{*}\frac{\rho_{N}^{*-2/3}}{\sqrt{T^{*}}}{\left(-s_{\mathrm{conf}}^{*}\right)}^{2/3}$$

7 $$D^{o}=D^{*}\frac{\rho_{N}^{*1/3}}{\sqrt{T^{*}}}{\left(-s_{\mathrm{conf}}^{*}\right)}^{2/3}$$

where η*, λ*, and D* denote the reduced shear viscosity, thermal conductivity, and self-diffusion coefficient, respectively. ρ_N_^*^ is the reduced number density, T* the reduced temperature, and *s*_conf_^*^ the reduced configurational entropy. Definitions of the reduced quantities are given in Table 4.

**Table 4:** Definition of the Reduced Physical Properties with the Reference Length *σ*_ref_, Reference Energy ε_ref_, and Reference Mass *m*_ref_

viscosity	$\eta^{*}=\frac{\eta \sigma_{\mathrm{ref}}^{2}}{\sqrt{\varepsilon_{\mathrm{ref}}m_{\mathrm{ref}}}}$	number density	ρ_N_^*^ = ρ_N_ σ_ref_^3^
thermal conductivity	$\lambda^{*}=\frac{\lambda \sigma_{\mathrm{ref}}^{2}}{k_{B}\sqrt{\frac{\varepsilon_{\mathrm{ref}}}{m_{\mathrm{ref}}}}}$	temperature	$T^{*}=\frac{T}{\left(\frac{\varepsilon_{\mathrm{ref}}}{k_{B}}\right)}$
self-diffusion coefficient	$D^{*}=\frac{D}{\sigma_{\mathrm{ref}}\sqrt{\frac{\varepsilon_{\mathrm{ref}}}{m_{\mathrm{ref}}}}}$	entropy	$s_{\mathrm{conf}}^{*}=\frac{s_{\mathrm{conf}}}{R}$

The resulting dimensionless, reduced and scaled properties are denoted as η^o^, λ^o^, and *D* ^o^. To describe the dilute gas limit, *Chapman-Enskog* expressions^83^ for the corresponding dimensionless, reduced transport properties are used:

8 $$\eta_{\mathrm{CE}}^{o}=\frac{5}{16\sqrt{\pi }}\frac{1}{\Omega^{\left(2,2\right)}}{\left(T^{*}\frac{dB^{*}}{dT^{*}}+B^{*}\right)}^{2/3}$$

9 $$\lambda_{\mathrm{CE}}^{o}=\frac{75}{64\sqrt{\pi }}\frac{1}{\Omega^{\left(2,2\right)}}{\left(T^{*}\frac{dB^{*}}{dT^{*}}+B^{*}\right)}^{2/3}$$

10 $$D_{\mathrm{CE}}^{o}=\frac{3}{8\sqrt{\pi }}\frac{1}{\Omega^{\left(1,1\right)}}{\left(T^{*}\frac{dB^{*}}{dT^{*}}+B^{*}\right)}^{2/3}$$

where Ω^(1,1)^ and Ω^(2,2)^ are the reduced collision integrals from Kim and Monroe,^84^ which are functions of the reduced temperature T*. The effective size parameter σ_CE_ is determined via the corresponding states principle,^85^ by mapping the Mie fluid to the *Lennard-Jones* reference substance. The reduced second virial coefficient B* and its temperature derivative $\frac{{dB}^{*}}{{dT}^{*}}$ were calculated analytically^24^ for the Mie fluid. Simple integration is possible here, due to the symmetry of the Mie particles, the presence of identical interaction partners, and the absence of multiple interaction types. Thus, B* was computed using rigorous statistical mechanics^86^ as

$$B^{*}\left(T^{*}\right)=2\pi \int_{0}^{\infty }f\left(r^{*},T^{*}\right)r^{*2}dr^{*}$$ 11

with r* = *r*/σ_ref_ being the distance between particles, and

12 $$f\left(r^{*},T^{*}\right)=1-\exp\left[\frac{-u_{\mathrm{Mie}}^{*}}{T^{*}}\right]$$

the Mayer f-function *f*(r*,T*), employing the Mie potential *u*_Mie_^*^ (cf. eq 1), and the reduced temperature T*. The temperature derivative of B* can be directly calculated by differentiating the Mayer f-function under the integral sign, leading to

$$\frac{dB^{*}}{dT^{*}}=2\pi \int_{0}^{\infty }\frac{df\left(r^{*},T^{*}\right)}{dT^{*}}r^{*2}dr^{*}=-\frac{2\pi }{T^{*2}}\int_{0}^{\infty }u_{\mathrm{Mie}}^{*}\exp\left[\frac{-u_{\mathrm{Mie}}^{*}}{T^{*}}\right]r^{*2}dr^{*}$$ 13

A second route to determine the second virial coefficient of the Mie fluids is by using an equation of state (EOS) (details given below).

The entropy scaling framework further distinguishes a low-density (LD) and high-density (HD) regime. The finally scaled transport property *Ŷ* ^o^ ∈{ η̂^o^, λ̂^o^, *D̂* ^o^ } is computed as a weighted sum

14 $${Ŷ}^{o}={Ŷ}_{\mathrm{LD}}^{o}W+{Ŷ}_{\mathrm{HD}}^{o}\left(1-W\right)$$

with the transition function *W* defined as

15 $$W=\frac{1}{1+\exp\left[20\left({\mathrm{s̃}}_{\mathrm{conf}}-{\mathrm{s̃}}_{\mathrm{conf}}^{\times }\right)\right]}$$

where *s̃*_conf_ = – *s*_conf_^*^ is the reduced configurational entropy, *s̃*_conf_^×^ = 0.5 is the crossover value.

The LD and HD contributions are normalized as

16 $${Ŷ}_{\mathrm{LD}}^{o}=\frac{Y_{\mathrm{LD}}^{o}}{Y_{\mathrm{CE}}^{o}},\mathrm{and}$$

17 $${Ŷ}_{\mathrm{HD}}^{o}=\frac{Y_{\mathrm{HD}}^{o}}{\min\left(Y_{\mathrm{CE}}^{o}\left(T\right)\right)}$$

The relation between the scaled transport coefficient *Ŷ* ^o^ and the configurational entropy *s̃*_conf_ is modeled with an empirical correlation:

18 $$\begin{array}{l} \ln\left[{\mathrm{\eta ̂}}^{o}\left({\mathrm{s̃}}_{\mathrm{conf}}\right)\right]\\ {\mathrm{\lambda ̂}}^{o}\left({\mathrm{s̃}}_{\mathrm{conf}}\right)\\ \ln\left[{\mathrm{D̂}}^{o}\left({\mathrm{s̃}}_{\mathrm{conf}}\right)\right] \end{array}\}=\frac{\alpha_{0}+\alpha_{\ln}\ln\left({\mathrm{s̃}}_{\mathrm{conf}}+1\right)+\alpha_{1}{\mathrm{s̃}}_{\mathrm{conf}}+\alpha_{2}{\mathrm{s̃}}_{\mathrm{conf}}^{2}+\alpha_{3}{\mathrm{s̃}}_{\mathrm{conf}}^{3}}{1+g_{1}\ln\left({\mathrm{s̃}}_{\mathrm{conf}}+1\right)+g_{2}{\mathrm{s̃}}_{\mathrm{conf}}}$$

where *g*_1_ and *g*_2_ are universal constants and α_*j*_ are in general component-specific parameters. In this work, the α_*j*_ parameters were obtained from a fit to the pure component Mie transport property computer experiment data (details given below). The numeric values are reported in the Supporting Information.

For viscosity and thermal conductivity, the entropy scaling model (cf. eq 18) can be directly applied for predicting mixture transport properties using the α_*j*_ parameters from the corresponding pure components. For this purpose, classical mixing rules are applied to the parameter vectors:

$$\begin{array}{c} {\underset{\_}{\beta_{\mathrm{mix}}\;}}^{\left(Y\right)}=\overset{L}{\underset{i}{\sum }}x_{i}{\underset{\_}{\beta_{i}\;}}^{\left(Y\right)},\\ {\underset{\_}{\beta_{i}\;}}^{\left(Y\right)}=\left(\alpha_{0,i}^{\left(Y\right)},\alpha_{\ln,i}^{\left(Y\right)},\alpha_{1,i}^{\left(Y\right)},\alpha_{2,i}^{\left(Y\right)},\alpha_{3,i}^{\left(Y\right)}\right) \end{array}$$ 19

Here, β_*i*_ denotes the component-specific parameter vectors for each of the *L* components, *x*_*i*_ represents the mole fraction of component *i*, and β_mix_ is the resulting mixture parameter vector obtained via linear mixing.

For modeling diffusion coefficients in binary mixtures, the extension proposed by Schmitt et al.^6^ was used. In this framework, the mixture is represented by limiting-case models consisting of pure-component self-diffusion models and infinite-dilution self-diffusion models, where the latter are treated as pseudo-pure component models. These limiting-case models are combined using the parameter-vector mixing rule given in eq 19. Thus, the *Maxwell–Stefan* mutual diffusion coefficient is obtained from the corresponding composition-dependent combination of the limiting self-diffusion models and the configurational entropy of the mixture.

Two sets of ES transport properties are considered in this work: (a) ES based solely on the computer experiment data (i.e., no EOS information was used); (b) transport coefficients computed using ES + EOS. (a) was primarily used for assessing the assumptions of ES itself, i.e., the scaling. (b) was primarily used for assessing the executable fluid-theory framework. For (a), the virial coefficients were computed from direct integration of the interaction potential; for (b), from the EOS – to maintain thermodynamic consistency.

#### Parameterization of Entropy Scaling Model

The entropy scaling correlation (cf. eq 18) contains the parameters α_*j*_, with *j* ∈ {0, ln, 1, 2, 3}. These parameters determine the relation between the scaled transport property *Ŷ* ^o^ and the configurational entropy *s̃*_conf_. In the present work, the α_*j*_ parameters were fitted to the pure component computer experiment data. For the viscosity and thermal conductivity, all five parameters were used, whereas for self-diffusion only α_2_ and α_3_ were used, which is primarily due to the fact that the functional form behind these two parameters provides a good description of the shape of ln **D̂* ^o^* (*s̃*_conf_). The target quantities for the fits were defined from the scaled transport properties, i.e., ln­(η̂^o^) for viscosity, λ̂^o^ for thermal conductivity, and ln­(*D̂* ^o^) for self-diffusion. The numerical values obtained from the fit and additional details on the parametrization procedure are given in the Supporting Information. For the thermal conductivity and the shear viscosity the transport property data from all considered Mie fluids collapse to a single scaling curve. Thus, a single α_*j*_ parameter set describes the thermal conductivity of all Mie fluids; and a single α_*j*_ parameter set describes the shear viscosity of all Mie fluids. For the self-diffusion coefficient, on the other hand, the scaling functions depend on the Mie exponents. Therefore, an empirical model was developed describing the α_*j*_ parameters as a function of the Mie exponents. Details are given in the Supporting Information. For mixture diffusion coefficients additional models describing the infinite dilution behavior need to be calibrated.

#### Molecular-Based Equations of State

The SAFT-VR Mie equation of state,^9^ which is based on Mie segment interactions, was employed in this work to describe the static thermodynamic properties of the studied pure Mie substances and Mie mixtures. Within the SAFT-VR Mie framework, the algebraic expression for the contact value of the radial distribution function of the Mie fluid is only valid for repulsive and attractive exponents in the range 5 ≤ λ_EOS_ ≤ 100.^9^ As a result, Mie fluids with exponent pairs (8, 4), (12, 4), and (20, 4) lie outside this validity range and cannot be represented within this EOS formulation. As a consequence, EOS-coupled entropy-scaling predictions based on the SAFT-VR Mie formulation were not generated for exponent pairs outside the valid parameter range of the EOS contact-value expression. Nevertheless, these fluids were analyzed on the basis of molecular simulation data via route (a).

### Evaluation Metrics

To quantify the accuracy of the fluid theory (route (b)) for describing the molecular simulation reference data, two metrics were used in this work: The average absolute deviation (AAD) and the median absolute deviation (MAD), defined as

$$\mathrm{AAD}=\frac{1}{N_{\mathrm{states}}}\overset{N_{\mathrm{states}}}{\underset{q=1}{\sum }}\left|\frac{Y_{\mathrm{mod},q}^{*}-Y_{\mathrm{sim},q}^{*}}{Y_{\mathrm{sim},q}^{*}}\right|$$ 20

21 $$\mathrm{MAD}=\mathrm{median}\left\{\left|\frac{Y_{\mathrm{mod},q}^{*}-Y_{\mathrm{sim},q}^{*}}{Y_{\mathrm{sim},q}^{*}}\right|\right\}$$

where *Y*_sim,*q*_ is the simulation data point *q*, *Y*_mod,*q*_ is the corresponding value calculated from the fluid-theory model, cf. eq 18, at the same T* and p* as the molecular dynamics simulation, and *N*_states_ is the number of states.

To quantify the collapse of the scaled transport property data to a monovariate curve, the standard deviation within each *s̃*_conf_ bin (SD_*b*_) was computed as

22 $${\mathrm{SD}}_{b}=\sqrt{\frac{1}{N_{b}}\sum_{q=1}^{N_{b}}{\left(\ln\left({Ŷ}_{q}^{o}\right)-\ln\left({Ŷ}_{\mathrm{med},b}^{o}\right)\right)}^{2}}$$

where *N*_*b*_ is the number of data points in bin *b* (defined over *s̃*_conf_), *Ŷ* ^o^_*q*_ denotes the *q*-th scaled data point of a transport property *Y* in that bin, and *Ŷ^o^*_med,*b*_ is the median of all *Ŷ* ^o^ values in bin *b*.

The corresponding 95% confidence intervals of SD_*b*_ were estimated by bootstrap resampling across substances. For each bootstrap replicate, substances were sampled with replacement within the respective evaluation group, SD_*b*_ was recomputed for each *s̃*_conf_ bin, and the 97.5th percentile of the resulting bootstrap distribution was reported as the upper confidence limit. In addition, a data-point-weighted mean standard deviation over all bins was computed, and its 95% confidence interval (CI) was obtained analogously from the bootstrap replicates.

## Results and Discussion

The results are discussed with respect to two main questions: (i) To what extent do the transport properties adhere to the entropy scaling principle, i.e., collapse to a monovariate function; (ii) What is the accuracy of the fluid theory for describing the reference data.

### Pure Components

Figure 5 shows the pure component transport properties using the scaled coordinates. Figure 6 shows the results for the bin-wise standard deviation from the monovariate function. Results are shown in both Figures 5 and 6 for the cases (a) and (b). This provides detailed insight into the applicability of the entropy scaling principle, i.e., properly scaled transport properties follow a monovariate function *Ŷ* ^o^ = *Ŷ* ^o^(*s̃*_conf_) and how these functions vary for different Mie fluids.

**Figure 5:**
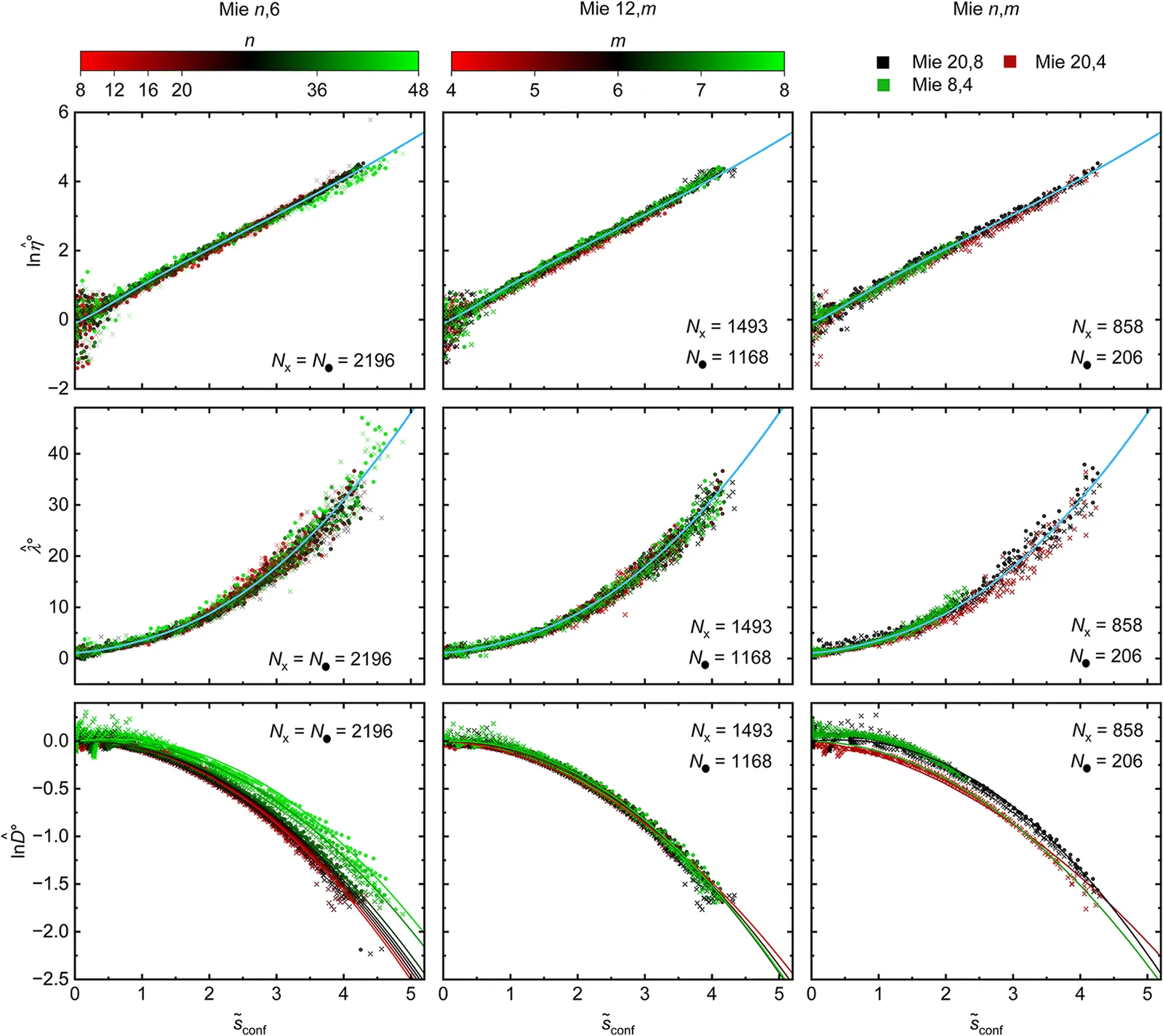
Entropy-scaling behavior for orthogonal and mixed Mie exponent families. Crosses denote results based on configurational entropies from molecular simulations combined with analytically calculated virial coefficients, whereas filled circles denote the corresponding results obtained using the molecular-based EOS. Solid lines represent the entropy-scaling model (cf. eq 18). In the left and middle panels, the color scale indicates the value of the Mie exponent varied within the respective family. Left: orthogonal family with fixed attractive exponent (*m* = 6) and repulsive exponents *n* ∈ {8, 10, 12, 14, 16, 20, 36, 48}. Middle: orthogonal family with fixed repulsive exponent (*n* = 12) and attractive exponents *m* ∈{4, 5, 6, 7, 8}. Right: selected mixed-exponent cases (Mie (20, 8), Mie (20, 4), and Mie (8, 4)). The labels in each panel report the number of cross-symbol data points (*N*_×_) and filled-circle data points (*N*_•_).

**Figure 6:**
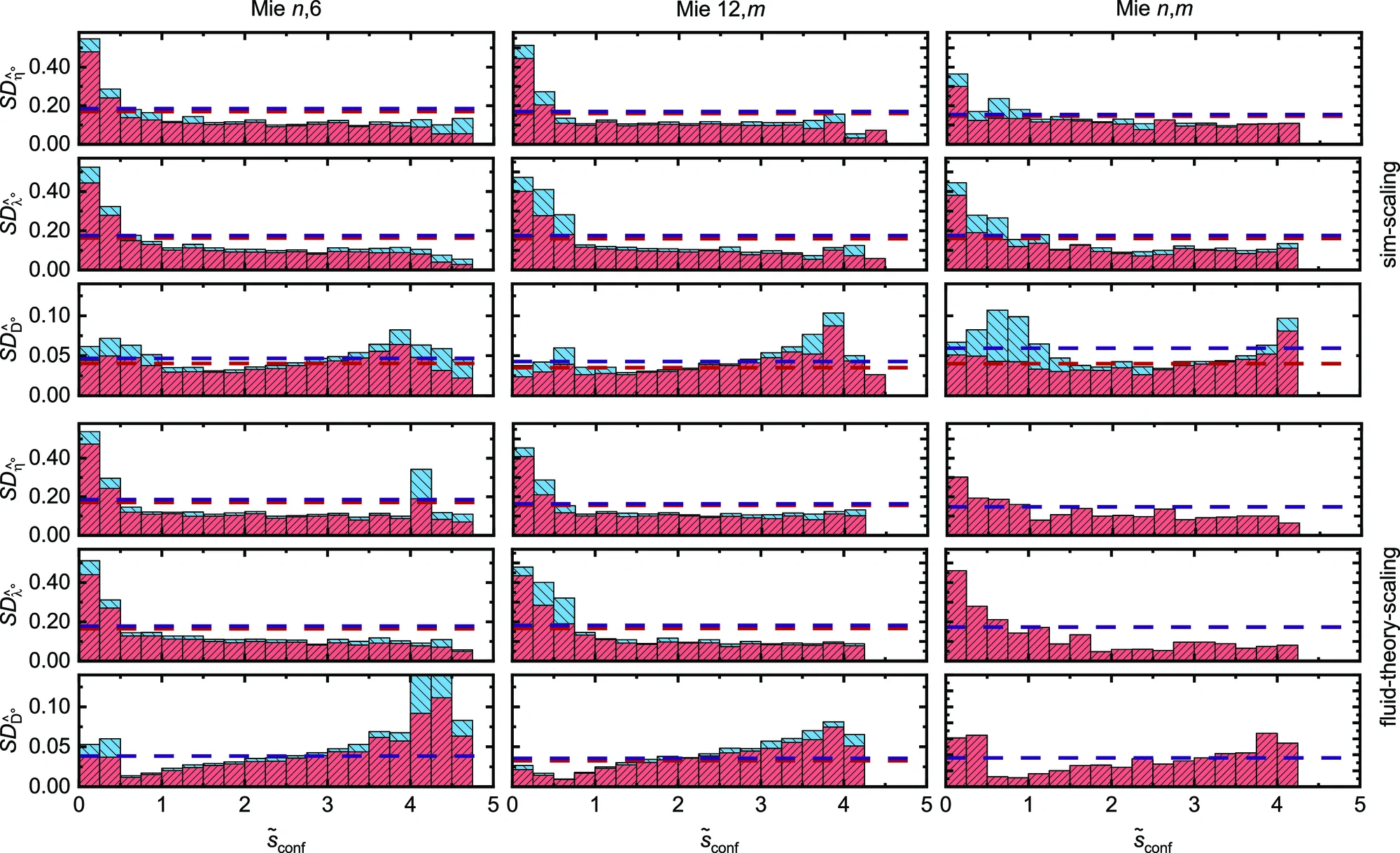
Standard deviations (cf. eq 22) for the scaled transport properties shown in Figure 5. Top: Scaling with entropy values from molecular simulations combined with analytically calculated virial coefficients (sim-scaling). Bottom: Scaling with molecular-based EOS entropy values and virial coefficients (fluid-theory-scaling). Red bars denote the standard deviation in each *s̃*_conf_ bin; blue bars indicate the corresponding 97.5% confidence percentile obtained by bootstrapping across substances. The red and blue dashed lines denote the data-point-weighted mean values over all bins for the red bars (SD) and blue bars (95% CI), respectively.

Overall, the results adhere to the entropy scaling principle for all studied Mie fluids and transport properties studied, however, not to the same degree: While the viscosity and the thermal conductivity data collapse onto a single monovariate function that is common to all studied Mie fluids, the self-diffusion coefficient requires an exponent-dependent parameterization, i.e., the scaling function is not universal within the Mie fluid class. Nevertheless, important differences are observed for different transport properties, different Mie fluid classes, and the different data sets (a and b), which are discussed in detail in the following. Overall, deploying the EOS for describing thermodynamic properties in the ES framework (i.e., route (b)) preserves the main entropy scaling structure.

Important differences are obtained for the three transport properties, cf. Figure 5: For the viscosity and the thermal conductivity, the transport properties from Mie fluids within a Mie fluid class as well as across the three studied Mie fluid classes ((*n*, 6), (12, *m*), and (*n*, *m*)) collapse to a single monovariate function. Thus, the model curves shown in Figure 5 were constructed according to the observed scaling behavior of the respective transport property. For the viscosity and thermal conductivity, a single entropy-scaling correlation was used for all investigated Mie fluids. For the self-diffusion coefficient, on the other hand, the function depends on the Mie potential exponents. Therefore, exponent-dependent correlations were developed. The exponent dependence of the self-diffusion scaling function and the systematic deviations observed for large repulsive exponents therefore constitute a limitation of the universality of entropy scaling within this model class, rather than a mere parameterization detail. The obtained parameters for the viscosity and thermal conductivity, as well as the hierarchical exponent-dependent parameterization for the self-diffusion coefficient, are given in the Supporting Information. For the Mie (*n*, 6) fluid class in the range 9 < *n* < 18, these results are in line with those reported by Saric et al.^38^ (who did not investigate 12, *m* and “mixed exponent” Mie fluids). In this work, we investigated a significantly larger range of *n* exponent values. Interestingly, for approximately *n* > 20, the scaling behavior covering different Mie fluids breaks for the self-diffusion coefficient, cf. Figure 5. This is similarly observed for “mixed exponent” Mie fluids for the self-diffusion coefficient, where both *n* and *m* cause a perturbation of the scaling function. For the viscosity and the thermal conductivity, the common monovariate scaling behavior covers all studied Mie fluids. This fundamental difference of the viscosity and the thermal conductivity on the one hand and the self-diffusion coefficient on the other hand is in line with differences in their topology.^48^

The influence of the Mie exponents – in particular of *n* – on the variation of the scaling functions can be furthermore characterized: Increasing *n* shifts the scaling curves to larger *s̃*_conf_ values. A systematic *n*-dependence of *s̃*_conf_ is already present at fixed (*T*, ρ), indicating that increasing the repulsive exponent primarily modifies the mapping (*T*, ρ) → *s̃*_conf_. A physically plausible interpretation is that increasing *n* steepens the repulsive core and increases the effective excluded-volume character, e.g., in terms of a larger *Barker-Henderson* effective hard-sphere diameter *d*_eff_(*T*).^87^ At constant (*T*, ρ) this increases the effective packing and strengthens short-range order (more pronounced peaks in the radial distribution function *g*(*r*)), which is associated with a more negative excess (i.e., configurational) entropy;^88,89^ since *s̃*_conf_ = – *s*_conf_^*^, this implies larger *s̃*_conf_. Consequently, the apparent right-shift of the entropy-scaling curves is predominantly thermodynamic in origin rather than a change in the functional form of the reduced transport-entropy relation. Consistently, simulation- and EOS-derived entropies agree closely, ruling out EOS bias as the origin of this trend. This indicates that states with comparable *s̃*_conf_ occur at different locations in the (*T*, ρ) plane when the Mie exponents are varied. This suggests that the apparent horizontal shift in the entropy scaling representation is related to an exponent-dependent thermodynamic mapping from (*T*, ρ) to *s̃*_conf_. Such an interpretation is consistent with the role of configurational entropy contours in isomorph theory^47,90^ and with previous entropy scaling studies of monatomic model fluids with variable repulsive interactions.^38^ The shape of the self-diffusion coefficient scaling function is, however, hardly distorted upon a Mie exponent variation. Thus, the effect of the Mie exponent can be captured easily in a heuristic approach – details on this approach are given in the Supporting Information.

For the different transport properties, different Mie fluids, and state regions, some scatter and minor deviations from the entropy scaling principle are observed, cf. Figure 5. Two types of deviations from the entropy scaling principle are observed: First, a visible random scatter of data points; second, smaller sets of data points slightly shift off in an apparently systematic manner. Deviations from the entropy scaling principle can decrease the accuracy of entropy scaling-based fluid-theory models. Thus, understanding deficiencies of the scaling is critical for a dedicated development of improvements in the future. Both Figures 5 and 6 provide insights into these deviations. For both the viscosity and the thermal conductivity, significant scatter is observed for low *s̃*_conf_ values – for all three studied Mie families. For the viscosity, this low density scatter is clearly visible in Figure 6; for the thermal conductivity, this behavior is largely obscured by the selected ordinate range. Figure 6 furthermore reveals that the normalized scattering behavior for viscosity and thermal conductivity is quantitatively very similar, i.e., relatively large scatter at low *s̃*_conf_ values. Since low *s̃*_conf_ corresponds to dilute, gas-like states, the affected points are predominantly located at the low densities (mostly at high temperatures) in the data set, where *Green–Kubo* estimates of η and λ tend to exhibit larger relative statistical uncertainty. Consistently, simulation- and EOS-derived entropies agree closely, indicating that the trend is not driven by the entropy evaluation.

With increasing *s̃*_conf_, the scatter decreases rapidly (cf. Figure 6). For *s̃*_conf_ ≈ 0.5–1.0, the scatter converges to approximately SD_*Y*_ ≈ 0.15 for both the viscosity and the thermal conductivity. For these two properties, no distinct systematic shifts of the data are observed; rather, the enhanced deviations at low *s̃*_conf_ are dominated by increased scatter (for the viscosity clearly visible in Figure 5, whereas for the thermal conductivity, it is largely masked by the ordinate scaling).

In contrast, for the self-diffusion coefficient, some systematic deviations from the monovariate scaling are observed (cf. Figure 5). Accordingly, SD_*D*_ follows a different pattern than SD_*Y*_ for the viscosity and thermal conductivity: Only slightly elevated values of SD_*D*_ are observed at low *s̃*_conf_, while SD_*D*_ is somewhat more increased at large *s̃*_conf_ (cf. Figure 6). The deviations at both low and high *s̃*_conf_ for *D* are largely driven by systematic offsets of groups of points and are most pronounced for large repulsive exponents *n* (cf. Figure 5). This behavior may be connected to the employed entropy scaling framework,^48^ which transitions from a low-density to a high-density branch at *s̃*_conf_ = 0.5 (cf. eq 14). In the low-density branch, the scaled diffusion data form approximately linear, isochoric clusters that are nearly perpendicular to the model curve; such isochoric clustering is also visible at larger *s̃*_conf_ and becomes more pronounced with increasing *n*. This might indicate that the employed *Chapman-Enskog* model has deficiencies at moderate densities before approaching the dilute gas limit.

Interestingly, these deviations are (except for the Mie (*n*, 6) family at large *s̃*_conf_) generally more pronounced for data set (a), i.e., when *s̃*_conf_ and the second virial coefficient are obtained directly from the potential. In contrast, for data set (b) – employing EOS-based inputs for *s̃*_conf_ and B* – the deviations are typically reduced, which is also reflected by the corresponding SD values in Figure 6. At first sight, it appears counterintuitive that introducing EOS-based thermodynamic inputs (which employs additional modeling assumptions) improves the apparent monovariate scaling behavior. We consider three plausible explanations: (i) compensating errors, i.e., EOS simplifications partially cancel deficiencies of the scaling formulation; (ii) the EOS provides a smoother, thermodynamically consistent mapping (T, ρ) → *s̃*_conf_, which can reduce residual numerical noise in the simulation-based entropy route even if the mean entropies agree closely; and (iii) the employed self-diffusion scaling form (eqs 7 and 10) may be less adequate for large *n* in the low-*s̃*_conf_ regime, consistent with the most pronounced systematic offsets observed in Figure 5.

Similar to the systematic shift deviations for low *s̃*_conf_ values at large *n*, shift deviations are also observed at high *s̃*_conf_ values, cf. Figure 5. At *s̃*_conf_ > 3, the diffusion data (most notably for the largest *n*) span a markedly broader band and exhibit clearer systematic offsets from the correlation (Figure 5). This behavior is consistent with a local distortion of the EOS-based mapping (T, ρ) → *s̃*_conf_ in this regime, which appears to overstretch the entropy coordinate and thereby amplifies the apparent separation of nearby state points in the diffusion scaling representation. The artifact is particularly visible for the self-diffusion coefficient because *D* varies more steeply with the entropy coordinate (in logarithmic representation) and because the diffusion data exhibit structured, isochoric groupings relative to the scaling curve; therefore, modest EOS-induced errors in *s̃*_conf_ can translate into noticeable clustering in the diffusion scaling plot.

The effect of introducing the EOS in the entropy scaling can also be assessed by comparing the top and bottom panels of Figure 6. Most importantly, introducing the EOS has stronger consequences for *D* compared to η and λ, indicating that the self-diffusion coefficient scaling is more sensitive to errors in the deployed entropy values *s̃*_conf_.

We have, furthermore, analyzed the scaling behavior in the different fluid state regions, including gaseous, liquid, supercritical, critical, and metastable states; the corresponding results are presented in the Supporting Information.

Figures 5 and 6 were primarily used to elucidate the applicability of the entropy scaling principle, i.e., the degree to which the transport property data yield a monovariate function for identifying deficiencies in individual model parts. The parity plots shown in Figure 7 focus on the accuracy of the overall fluid-theory model (ES + EOS) for describing the computer experiment data. For the three transport properties, the fluid theory yields AAD values of 15.8, 17.2, and 8.8% for describing the computer experiment reference data for the viscosity, thermal conductivity, and the self-diffusion coefficient, respectively. The parity analysis (cf. Figure 7) confirms that the fluid-theory model describes the MD reference data with good overall accuracy. Deviations from the parity line in the entropy panel propagate into the transport-property panels in a property-dependent manner, but the overall agreement supports the use of EOS-derived entropy values for fluid-theory entropy scaling within the validated exponent range of the EOS. This is particularly relevant for practical applications, because the EOS route avoids the need for direct molecular simulation of the entropy at each state point while retaining trends in the scaled transport-property predictions.

**Figure 7:**
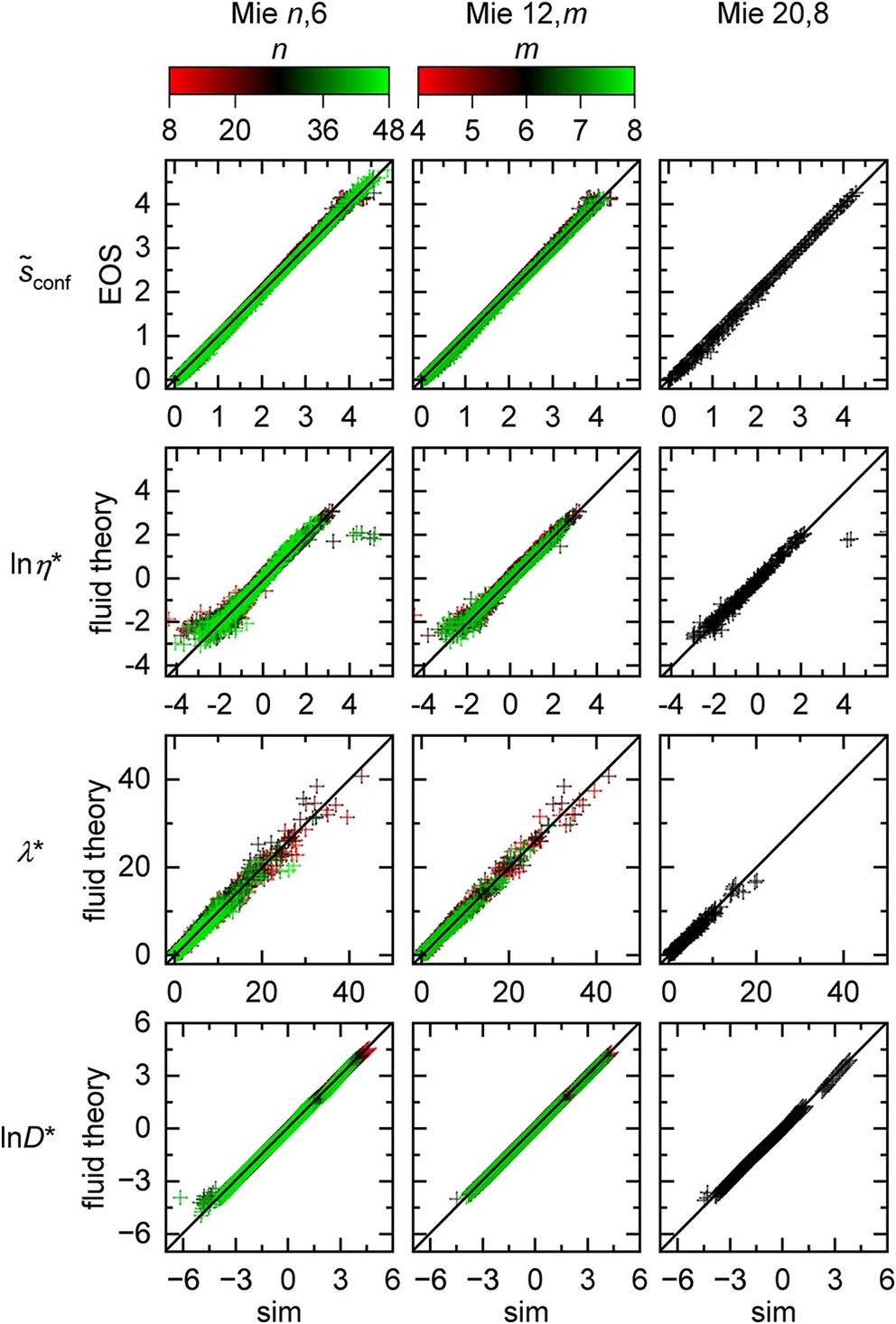
Parity plots for the configurational entropy and the reduced transport properties. The first row shows the parity plot for the configurational entropy from the EOS vs simulation data. Rows two to four show the parity plots for the reduced viscosity, thermal conductivity and self-diffusion coefficient obtained from the fluid-theory models vs the molecular simulation reference data. In the left and middle panels, the color scale indicates the value of the Mie exponent varied within the respective family.

At the same time, the parity plots provide a direct visualization of the limits of the fluid theory: Any systematic entropy bias relative to the reference data will translate into a shifted position along the entropy axis and, consequently, into a corresponding shift in the predicted reduced transport properties. The most obvious deviations occur for the thermal conductivity, where the fluid theory systematically underestimates the reference data. For the self-diffusion coefficient, most of the scattering occurs at high values, which correspond to states with low *s̃*_conf_. A comparison between the scaled reduced transport properties from both scaling methods (route a and b) is given in the Supporting Information.

### Binary Mie Mixtures

Mixture transport properties do not obey the entropy scaling principle the same way as pure components – the scaled transport properties of different pure components do not necessarily collapse onto a common monovariate function. This behavior has been observed, for example, for many real substance classes^48^ and the self-diffusion coefficient of Mie (*n*, 6) fluids. In the ES + EOS fluid-theory framework, mixtures are therefore represented by a one-fluid picture: The composition-dependent entropy scaling parameters are obtained from the mixing rules for the α parameters (cf. eq 19), and the mixture is evaluated as an effective pseudo-pure fluid. Figure 8 illustrates this construction for mixture B in the scaled coordinates.

**Figure 8:**
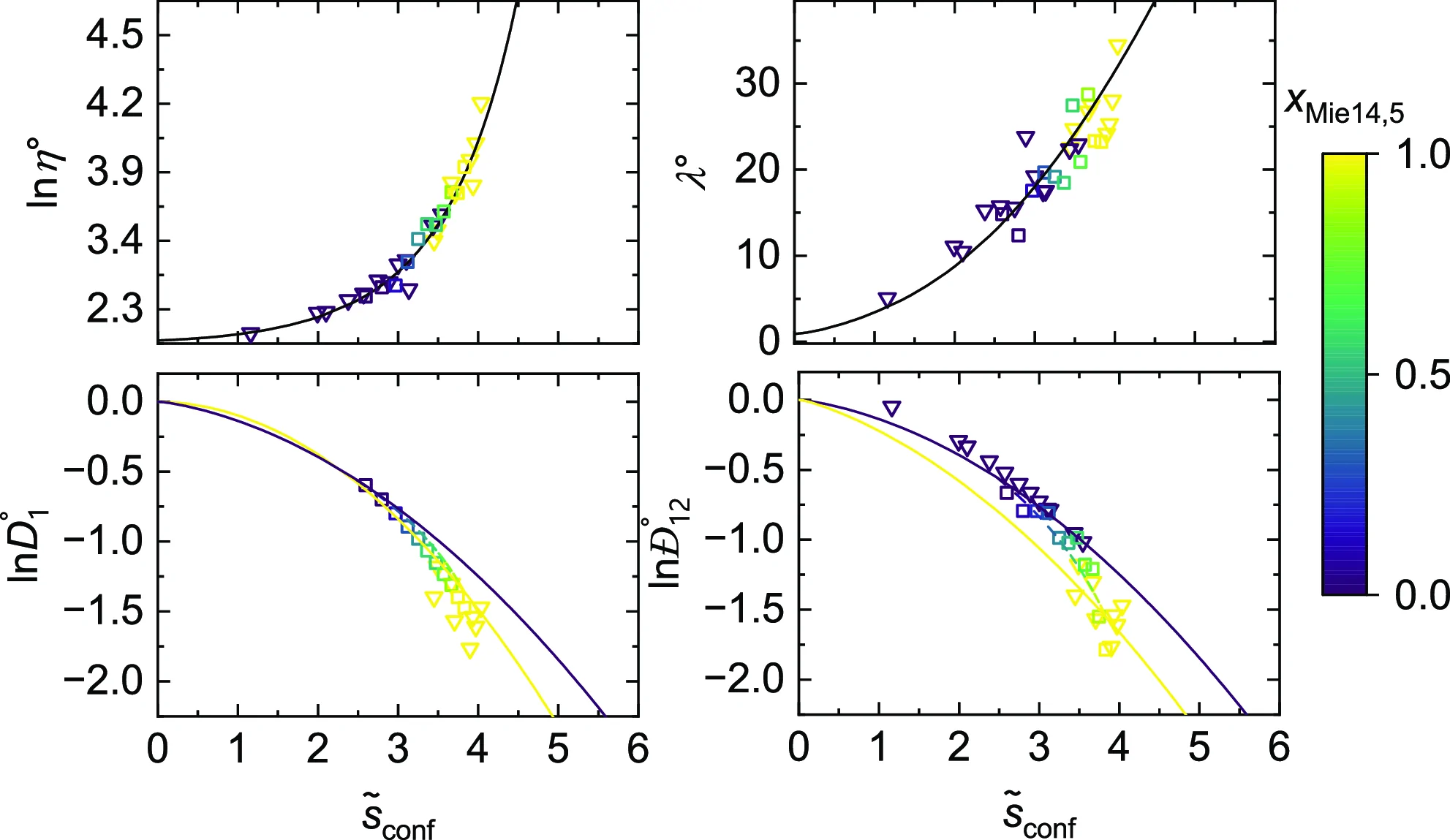
Entropy scaling representation of scaled transport properties of binary mixture B as functions of the configurational entropy *s̃*_conf_ (cf. eqs 18 and 19). Symbols denote scaled MD data. Open triangles indicate the pseudo-pure composition limits, whereas open squares indicate true mixture data along the isothermal–isobaric path at T* = 0.77 and p* = 0.1 across the full composition range. The color encodes the mole fraction *x*_Mie (14, 5)_. Solid lines show the entropy-scaling correlations of the (pseudo-)­pure component limits. Dashed lines show the composition-dependent mixture correlations evaluated along the same isothermal–isobaric path.

In Figure 8, the solid curves mark the entropy-scaling correlations for either all mixture compositions (viscosity and thermal conductivity) or the limiting cases (pure component or pseudo-pure component) for diffusion coefficients, whereas the dashed curves represent the composition-dependent mixture model along the selected isothermal–isobaric path. The mixture fluid-theory model provides a continuous transition between the limiting-case curves as the composition changes. The scaled MD mixture data follow these dashed curves rather than either pure-component limit alone, which supports the use of the α-parameter mixing rule to represent composition effects within the one-fluid picture. At the same time, the mixture data do not collapse onto a single universal curve: The position in *s̃*_conf_ and the scaled transport coefficient both shift with composition. This is particularly visible for the diffusion coefficients and reflects the fact that species mobilities and interspecies friction remain sensitive to composition and unlike interactions, even after entropy scaling.

The binary-mixture results provide a stringent test of transferability for the entropy-scaling framework because they introduce unlike interactions, composition dependence, and mixing-rule effects that are absent in pure fluids. For mixture A both components are part of the pure-fluid fitting set, such that mixture A tests the mixture model alone. In addition, mixture B (Mie (14, 5) + Mie (20, 7)) constitutes an out-of-sample transfer test: Both pure component exponent pairs lie outside the set of pure Mie fluids used to fit the hierarchical exponent-dependent parameterization. Consequently, predictions for transport properties in mixture B probe not only mixture transferability, but also the ability of the pure-fluid parameter correlations to generalize to previously unseen exponent combinations. For the viscosity and thermal conductivity, the common pure-fluid entropy scaling parameter sets reported in the Supporting Information were used without adjustment to mixture data. For the diffusion-related mixture predictions, the pure-component self-diffusion parameters were obtained from the exponent-dependent self-diffusion parameterization (fully predictive transfer). In addition, the mixture self-diffusion coefficients require infinite-dilution correlations following the pseudo-pure component interpretation.^6^ Therefore, an entropy scaling function was parametrized for each infinite dilution self-diffusion coefficient. The corresponding parameters were calibrated from mixture simulation data at *x*_*i*_ ≈ 0.001 mol·mol^–1^ (left edge for ln *D*_1_^*,∞^ and right edge for ln *D*_2_^*,∞^). The resulting pseudo-pure-component α parameters are reported in the Supporting Information. The self-diffusion coefficient at mixture states was then predicted by “mixing” the pure component entropy scaling model with the model for the corresponding infinite dilution. This distinction is important for interpreting the predictive scope of the framework and for comparing the performance across transport properties.

The viscosity and thermal conductivity mixture results are shown in Figures 9 and 10 in nonscaled variables. Overall, the entropy scaling model captures the influence of composition, temperature, and pressure well. This is remarkable considering the fact that the fluid theory results are predictions that have not been adjusted to any mixture data. In most cases, the two independent methods agree within the statistical uncertainties of the simulation results. Some scattering is observed in the simulation data for the viscosity and thermal conductivity, which is attributed to sampling effects.

**Figure 9:**
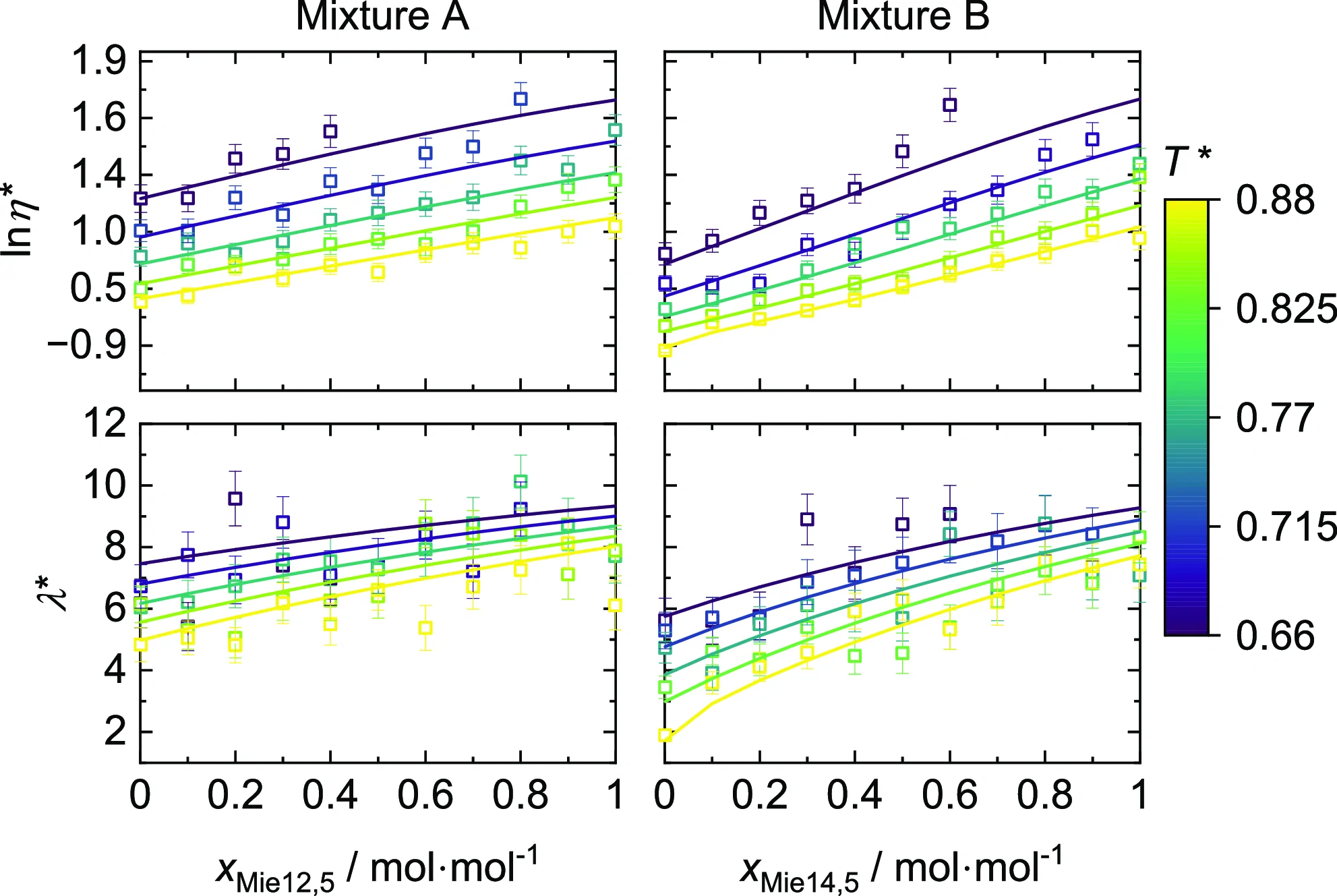
Viscosity and thermal conductivity for the mixtures A (Mie (12, 5) + Mie (16, 6)) and B (Mie (14, 5) + Mie (20, 7)) for p* = 0.1. The color indicates the five different reduced temperatures T* ∈ {0.66, 0.715, 0.77, 0.825, 0.88}. Symbols indicate simulation data, and lines represent predictions from the ES + EOS fluid-theory framework described in eqs 18 and 19.

**Figure 10:**
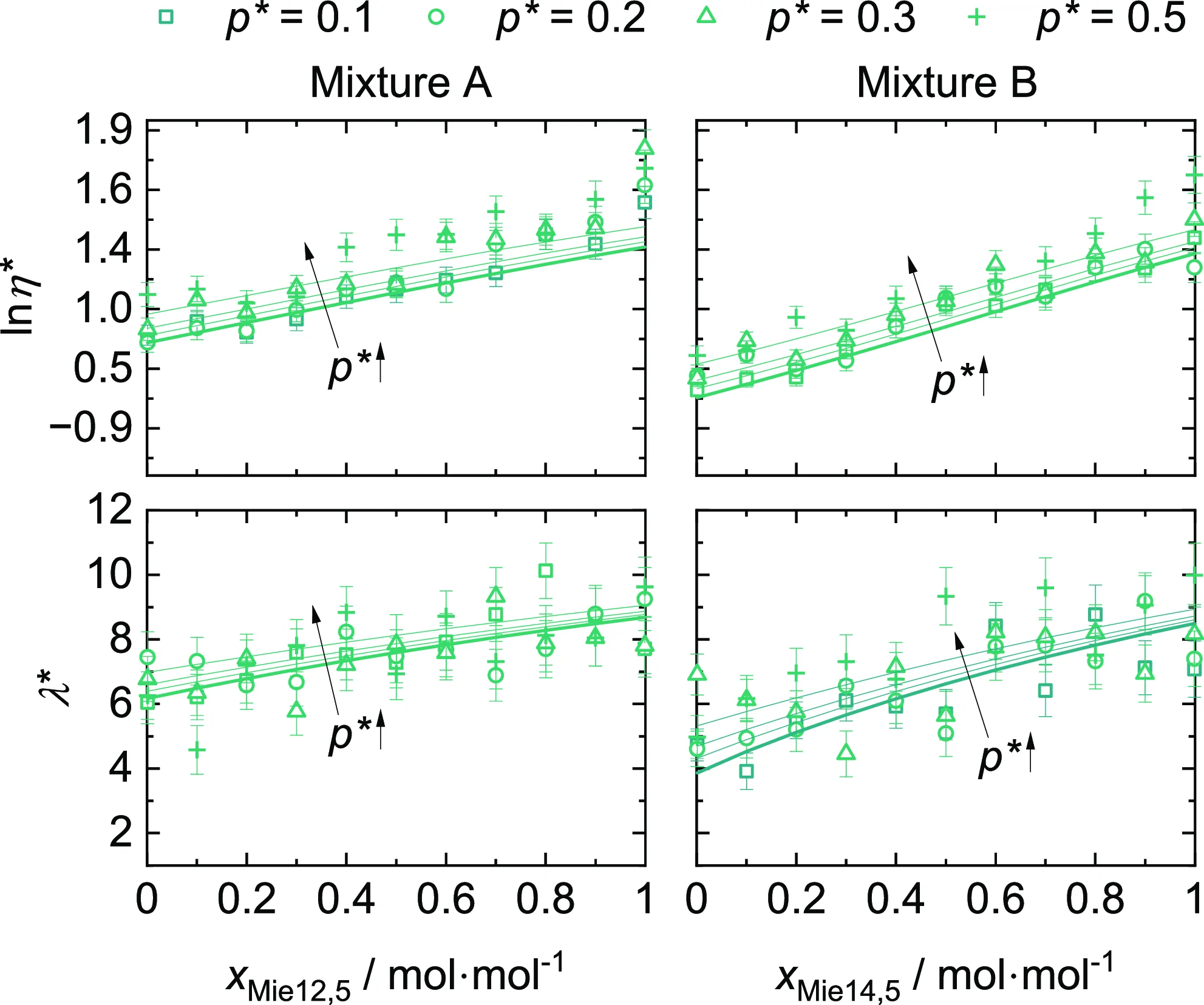
Viscosity and thermal conductivity for the mixtures A (Mie (12, 5) + Mie (16, 6)) and B (Mie (14, 5) + Mie (20, 7)) for T* = 0.77. The symbols indicate the four different reduced pressures p* ∈ {0.1, 0.2, 0.3, 0.5}. Symbols indicate simulation data, and lines represent predictions from the ES + EOS fluid-theory framework described in eqs 18 and 19.

Figure 11 shows the results for the three mixture diffusion coefficients (*D*_1_, *D*_2_, *Đ*_12_) for mixtures A and B at p* = 0.1. The corresponding results for other pressure values are shown in the Supporting Information. The accuracy of the fluid-theory predictions is overall very good – also in comparison to the viscosity and thermal conductivity mixture results discussed above. The effect of temperature, pressure, and composition is well captured by the fluid-theory predictions. This is partly due to the fact that the reference data exhibit very little scatter and small uncertainties. The fact that the uncertainties are very small enables the identification of areas, where the fluid-theory predictions do not agree with the reference data within their error bars, e.g., for *D*_2_ for mixture B at high *x*_Mie (14,5)_ values. Nevertheless, the fluid-theory model predicts all three diffusion coefficients very well over the full composition range – also for mixture B, which shows strong nonideal mixing effects.

**Figure 11:**
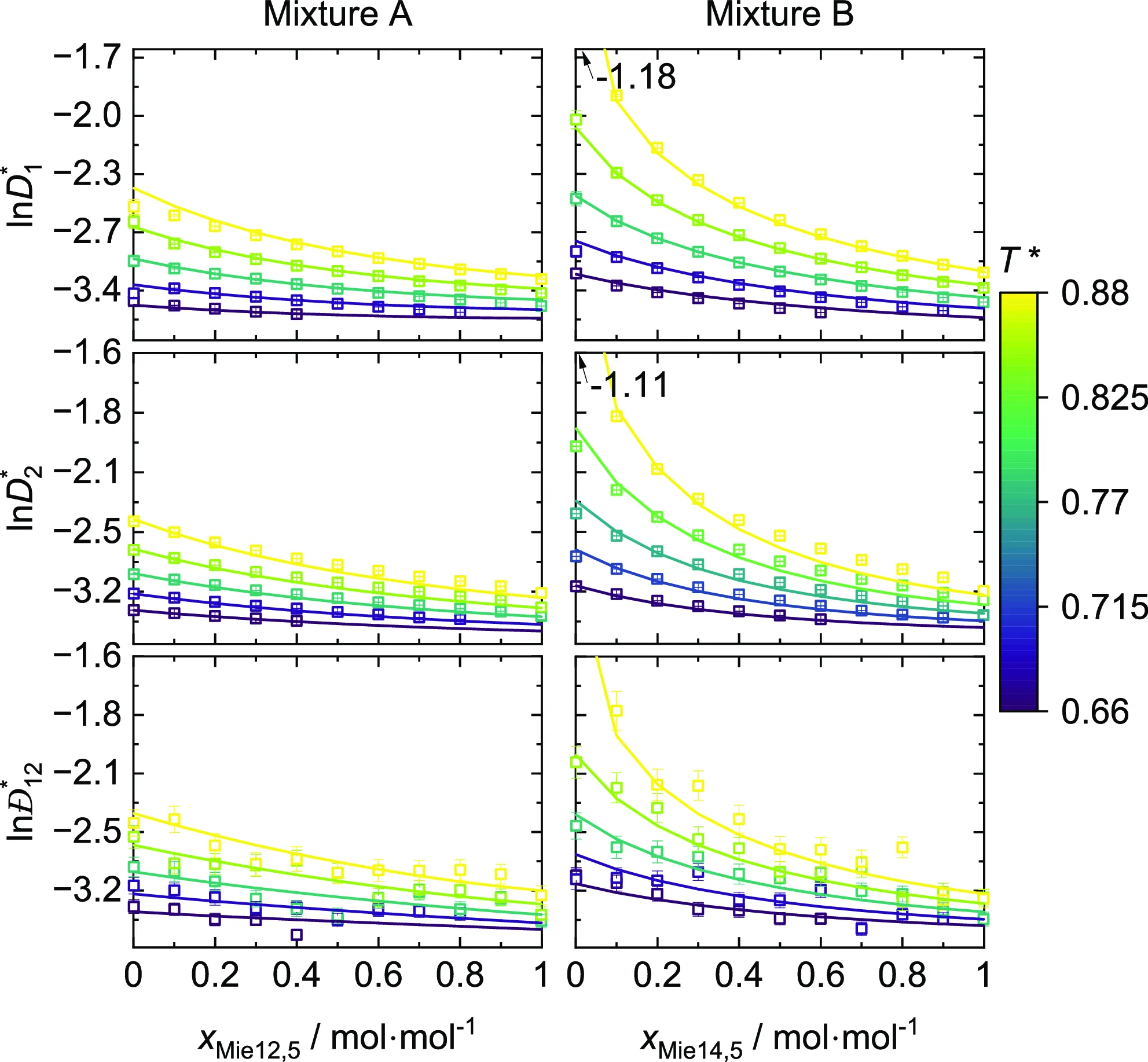
Diffusion coefficients for the mixtures A (Mie (12, 5) + Mie (16, 6)) and B (Mie (14, 5) + Mie (20, 7)) for p* = 0.1. The color indicates the five different reduced temperatures T* ∈ {0.66, 0.715, 0.77, 0.825, 0.88}. Symbols indicate simulation data, and lines represent predictions from the ES + EOS fluid-theory framework described in eqs 18 and 19.

The mean accuracy of the mixture fluid-theory predictions is compared in Table 5 to the corresponding values obtained for the pure components. Figure 12 shows the parity plots for the five studied mixture transport properties. For the pure components, the reported AAD and MAD values are the result of a fit, cf. Figure 5.

**Figure 12:**
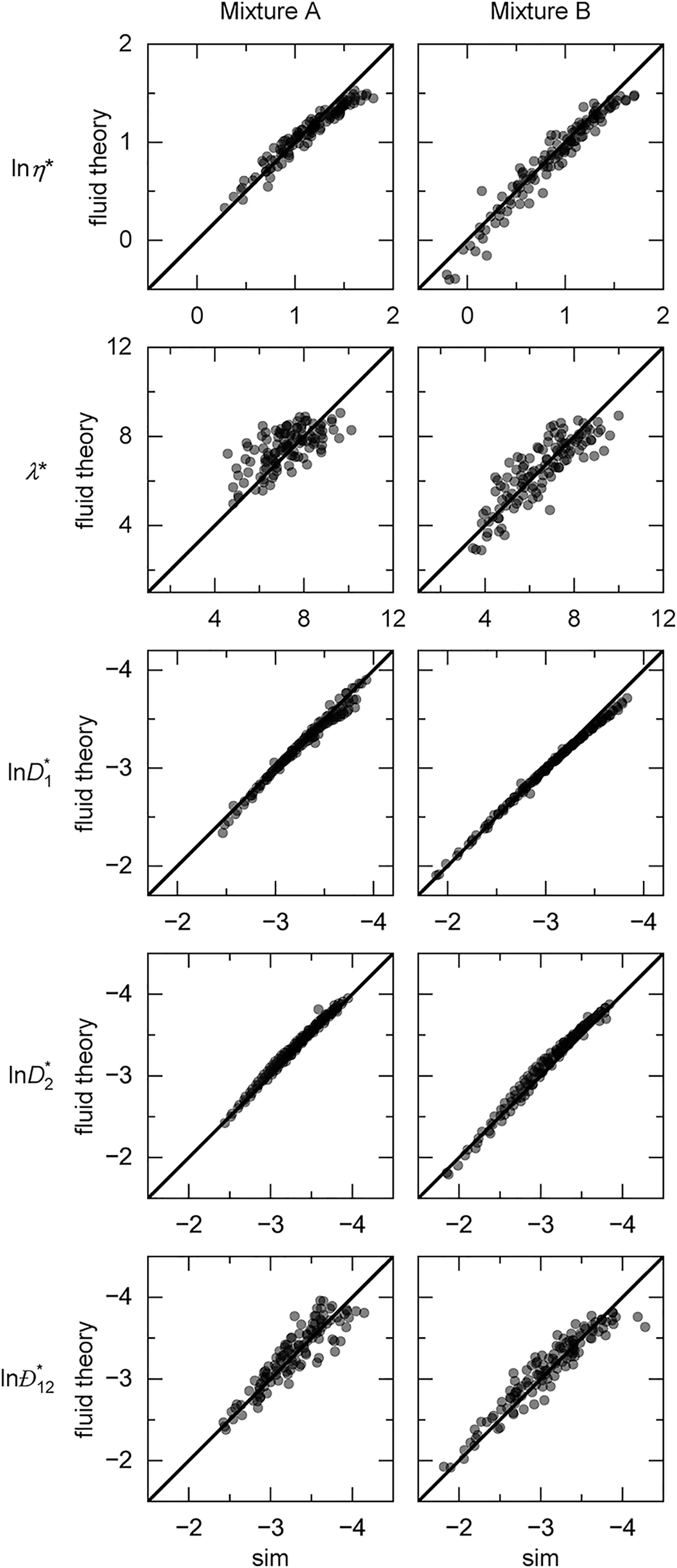
Parity plots for the reduced viscosity, thermal conductivity, and diffusion coefficients simulation data (sim) vs fluid-theory predictions for the two binary mixtures.

**Table 5:** Average Absolute Deviation (AAD) and Median Absolute Deviation (MAD) Error Measures (cf. eqs 20, 21) for the Fluid-Theory Model Compared to the MD Reference Data for the Mie (12, 6) Fluid, all Mie Fluids, Mixture A and Mixture B^a^

	Mie (12, 6)		Mie (*n*,*m*)		mixture A		mixture B	
property	AAD	MAD	AAD	MAD	AAD	MAD	AAD	MAD
η*	10.4	5.7	15.8	6.8	8.1	7.1	8.6	7.3
λ*	13.3	6.6	17.2	7.5	10.9	8.3	10.4	8.7
*D*_1_^*^	2.2	2.0	8.8	1.9	4.3	2.7	3.5	2.2
*D*_2_^*^					4.9	4.6	7.1	7.0
*Đ*_12_^*^					14.1	10.8	12.2	10.5

a For the pure components, the AAD and MAD values quantify the correlation accuracy of the fit; the mixture values are predictions. All values are given in %.

The comparison between pure-fluid and mixture results shows that the transition from pure components to mixtures does not lead to a systematic loss of accuracy for the properties studied here. For η and λ, the mixture AAD values are lower than the corresponding values for the full pure-Mie data set and are close to the values obtained for the Mie (12, 6) reference fluid. This is possibly due to the fact that a somewhat larger range of states was studied for the pure components. Thus, the approximations introduced in the mixing model retain good predictive capability for these two transport properties, although the parity plots reveal more scatter for the mixture thermal conductivity than for the pure-fluid thermal conductivity. For the diffusion coefficients, the two self-diffusion coefficients remain among the most accurately described mixture properties. The lowest deviations are obtained for *D*_1_, whereas the deviations for *D*_2_ are somewhat larger, especially for mixture B. The largest mean deviations are obtained for the *Maxwell–Stefan* diffusion coefficient, which indicates that the interspecies transport coefficient is more challenging to describe than the corresponding single-particle mobilities. Nevertheless, the mean accuracy values obtained for the *Maxwell–Stefan* diffusion coefficient are only moderately larger compared to those for the thermal conductivity. This supports the validity of the mixture diffusion coefficient framework developed in ref 6.

The comparison of the accuracy of the fluid theory for predicting the different mixture transport properties reveals that the best results are obtained for the two self-diffusion coefficients; the largest mean deviations are obtained for the *Maxwell–Stefan* diffusion coefficients. The relatively large mean deviations obtained for *Đ*_12_, λ, and η can be attributed to some extent to the scatter of the reference data, cf. Figures 9, 10, and 11. This is also supported by the scattering visible in the parity plots, cf. Figure 12. In general, the parity plots provide a complementary view of the model performance across the full mixture data set. Systematic deviations are only observed in the parity plots for *D*_1_ and η, where the fluid-theory predictions slightly underestimate the reference data for high transport coefficient values – especially for mixture A. In agreement with the AAD/MAD results, the self-diffusion coefficients show the closest clustering around the parity line, whereas the *Maxwell–Stefan* diffusion coefficients exhibit a broader spread. Also for the thermal conductivity mixture data, significant scatter is observed in the parity plot, cf. Figure 12. In particular, the scattering for the mixture thermal conductivity is significantly larger compared to the scattering observed in the pure component thermal conductivity parity plot, which is primarily due to the sampling in the MD simulations.

The different accuracy levels for the mixture transport properties can be rationalized by the different physical character of the corresponding transport mechanisms. The self-diffusion coefficients are single-particle properties and primarily reflect the mobility of the individual species in the mixture environment; hence, they are comparatively well described by the entropy coordinate and the species-specific scaling relations. In contrast, the *Maxwell–Stefan* diffusion coefficient is a collective interspecies transport property that depends explicitly on the relative motion and friction between sets of particles of the two components. It is therefore more sensitive to unlike interactions, composition-dependent nonideality, and cross-correlations in the mixture dynamics, which are only indirectly represented in a monovariate entropy scaling framework. This provides a plausible explanation for the larger deviations of the fluid-theory predictions for *Đ*_12_ compared to *D*_1_ and *D*_2_. In addition, part of the apparent fluid-theory deviation for *Đ*_12_, λ, and η can be attributed to the scattering of the MD reference data. These quantities are collective transport properties and are generally more susceptible to statistical noise than self-diffusion coefficients, because they are obtained from collective flux or relative-motion correlations. Consequently, the deviations in the parity plots reflect both limitations of the entropy scaling model and the uncertainty of the underlying reference data.

The comparison of AAD and MAD further indicates that the error distributions are property dependent. For η and *D*_2_, the AAD and MAD values are relatively close for both mixtures, indicating comparatively uniform deviations. For λ, *D*_1_, and *Đ*_12_, however, the AAD values exceed the corresponding MAD values significantly. This indicates that a subset of state points contributes disproportionately to the mean deviation. For λ and *Đ*_12_, this is consistent with the relatively broad scattering visible in the parity plots (cf. Figure 12) and in the composition-dependent representations (cf. Figures 9–11). For *D*_1_, the MAD remains small, but the larger AAD shows that a few higher-deviation state points are present despite the generally close clustering around the parity line.

## Conclusions

In this work, we systematically applied entropy scaling to different Mie fluids and Mie fluid mixtures for elucidating the behavior and applicability of the theoretical framework. In a first step, a large range of Mie fluids was studied to quantify the applicability of the entropy scaling principle, i.e., to what extent a monovariate function is formed. This assessment is based on a comprehensive new molecular dynamics data set – covering all fluid state regions. For the viscosity and the thermal conductivity, all data for a given transport property for all Mie fluids collapse to a single monovariate function. For the self-diffusion coefficient, on the other hand, the monovariate functions of different Mie fluids vary – especially for large values of the repulsive exponent *n*. For describing the influence of the Mie exponents on the self-diffusion coefficient entropy scaling parameters, an empirical heuristic model was developed. Consequently, the universality of the entropy scaling principle is limited for the self-diffusion coefficient. The empirical exponent-dependent model developed here yields a practically useful description, but it does not remove the underlying dependence of the scaling function on the interaction potential. Within the investigated range, the repulsive exponent has a stronger influence on the scaling behavior compared to the attractive exponent.

The scaled transport-property data collapse onto monovariate functions in nearly all cases. This includes metastable states and high-density states as well as supercritical states. Only for some extreme Mie exponent cases, some deviations from the entropy scaling principle were observed. Thus, the collapse to a monovariate function by the applied entropy scaling is consistent with an isomorph-motivated picture, but it does not by itself verify isomorph invariance. Nevertheless, the results from this work are a reminder that entropy scaling goes hand in hand with assumptions that are not fulfilled in all cases, which can lead to inaccuracies of an executable fluid-theory model.

Most importantly, the results show that entropy scaling fluid theory can yield predictions for mixture transport properties that agree in most cases with reference data within their uncertainties. This holds for all ranges of temperature, pressure, and composition that were studied in this work. This agreement is notable because no finite-composition mixture data were used to parameterize the model for mixture predictions. For the viscosity and thermal conductivity, the mixture results are pure predictions obtained directly from the common pure-fluid entropy-scaling parameter sets with no mixture-adjusted parameters. For the three diffusion coefficients (two self-diffusion and the *Maxwell–Stefan* diffusion coefficient) in the mixtures, infinite-dilution models were additionally parameterized following the approach proposed in ref 6. Then, the finite-composition mixture states were predicted using mixing rules of the entropy-scaling framework and the underlying EOS. The agreement of the molecular simulation results and the independent predictions from fluid theory for mixture B is particularly noteworthy, because it demonstrates the generalization to a binary system not included in the finite-composition parameterization. The strong predictive capabilities of the entropy scaling fluid theory from previous works of our group^6,48^ are a result of the connection between a physically-based Helmholtz energy model with a robust, physically motivated entropy scaling relation. The obtained physics-based model enables the traceable description of transport properties across all fluid regions by very few adjustable parameters, i.e., 2–5 for Mie fluid pure components and no additional parameters for viscosity and thermal conductivity in mixtures.

The results from this work indicate specific minor shortcomings of the entropy scaling framework, e.g., noticeable scatter in the dilute-gas regime, exponent-dependence of the self-diffusion scaling for steep and mixed-exponent Mie fluids, and remaining deviations for *Maxwell–Stefan* diffusion coefficients. These findings identify clear targets for future development of the framework. Moreover, a systematic comparison of different ES frameworks would be interesting for a future work.

## Supplementary Material









## Data Availability

All primary data obtained from the MD simulations are provided in the Supporting Information in a spreadsheet file. An explanation of that data is given in the Supporting Information PDF file.

## Notes

Mie fluid ES pure (XLSX)
Mie fluid GE (XLSX)
Mie fluid mix (XLSX)
Thermodynamic Integration; entropy scaling model parameters; GE simulations; number of state points studied by MD; pure component results; scaling behavior of different fluid state regions; binary Mie-mixture results; and modeling transport properties of Mie fluids (PDF)
