# Supplementary material for: Transport Properties of Mie Fluids: Molecular Dynamics Simulations and Entropy Scaling
Source: J Phys Chem B. 2026 Oct 15;130(43):11552–67. doi: 10.1021/acs.jpcb.6c04595 (PMC13726387; doi:10.1021/acs.jpcb.6c04595)
Supplement: Supplementary file 1 [file jp6c04595_si_004.pdf]

Supporting Information: Transport Properties of Mie Fluids: Molecular Dynamics Simulations and Entropy Scaling

Dennis Alt[†] and Simon Stephan[*,†,‡]

†*Molecular Thermodynamics Group (MTG), RPTU Kaiserslautern, Kaiserslautern 67663, Germany*

‡*Laboratory of Heat and Mass Transfer (WSÜ), OVGU Magdeburg, Magdeburg 39106, Germany*

E-mail: simon.stephan@ovgu.de

Contents of the Supporting Information

This Supporting Information contains additional methodological details, parameter tables, and supplementary figures supporting the main manuscript. The contents are summarized below.

- **Thermodynamic Integration:** Parameters used for the thermodynamic-integration calculations.

- **Entropy Scaling Model Parameters:** Parameter sets for the EOS-consistent ES + EOS framework including common entropy scaling parameters for η and λ as well as hierarchical exponent-dependent parameterization for D and entropy scaling parameters for infinite-dilution self-diffusion models.

- **GE Simulations:** Simulation procedure and fitting equations used to determine the critical properties of the mixed-exponent fluids.

- **Number of State Points Studied by MD:** Number of retained simulation data points after preprocessing and filtering.

- **Pure Component Results:** Additional pure-fluid parity plots.

- **Scaling Behavior of Different Fluid State Regions:** Additional plots per state region.

- **Binary Mie-Mixture Results:** Additional mixture results for viscosity, thermal conductivity, self-diffusion, *Maxwell–Stefan* diffusion, and configurational entropy.

- **Modeling Transport Properties of Mie Fluids:** Different modeling routes distinguished by their physical assumptions, applicability and accuracy.

Contents of the Electronic Supplementary Material

In addition to this PDF document, the Electronic Supplementary Material contains the numerical data generated and used in this work.

- `elec_supp_material_mie_fluid_ES_pure.xlsx`: This includes the molecular-simulation results for the pure Mie fluids together with both the sim-scaling and the fluid-theory-scaling entropy scaling results.
- `elec_supp_material_mie_fluid_mix.xlsx`: This includes the molecular-simulation results for the binary mixtures, thermodynamic factor from the EOS and the calculated Fickian diffusion coefficients.
- `elec_supp_material_mie_fluid_GE.xlsx`: This includes the primary VLE data from the GE simulations.

Thermodynamic Integration

The parameters used in the MD simulations for the thermodynamic integration method are given in Table S1.

Table S1: Parameters for the thermodynamic integration method.

NTest	12288
LambdaMin	0.2
NBins	100
LambdaStepMax	0.1
LambdaExponent	0.25

Entropy Scaling Model Parameters

This section summarizes the fitting procedure and results for the entropy scaling models. The parameters in Tables S2–S4 correspond to the EOS-consistent ES+EOS route used for the executable fluid-theory predictions.

Viscosity and Thermal Conductivity Parameters

The entropy scaling model parameters for the viscosity and the thermal conductivity for the Mie fluids are given in Table S2.

Table S2: Parameters for the entropy scaling model (cf. Eq. 18 in the main body of the text) for the viscosity and the thermal conductivity.

Quantity	α_0	$\alpha_{\ln}$	α_1	α_2	α_3
$\ln\left[\hat{\eta}^{\circ}\right]$	-0.09895	0.556788	0.30572	0.591216	0.071201
$\hat{\lambda}^{\circ}$	1.096097	-17.895	17.51415	-5.28638	1.732817

Self-Diffusion Parameters

This section provides details on the empirical model used to describe the dependence of the self-diffusion entropy-scaling parameters on the Mie exponents (n, m). The hierarchical fit

was applied only to the self-diffusion model. For viscosity and thermal conductivity, one common entropy scaling parameter set was used for all investigated Mie fluids, as reported above.

Hierarchical fit

The aim of the hierarchical fit is to replace separately fitted entropy scaling parameter sets for individual Mie fluids by a smooth exponent-dependent representation. This enables interpolation of the entropy-scaling parameters within the investigated Mie exponent space and allows predictions for Mie fluids whose exponent pairs were not part of the individual pure-fluid fitting set.

The entropy scaling model in the main manuscript describes the finally scaled self-diffusion coefficient $\hat{D}^{\mathrm{o}}$ as a function of the configurational entropy variable $\tilde{s}_{\mathrm{conf}}$. For the hierarchical fit, the self-diffusion target variable $y^{(D)}$ was defined from $\hat{D}^{\mathrm{o}}$ using a logarithmic transformation:

$$y^{(D)} = \ln\left(\hat{D}^{\mathrm{o}}\right). \tag{S1}$$

This choice follows the form of the entropy scaling model in Eq. (18) of the main manuscript.

We consider K pure-fluid data sets. Each data set k corresponds to one Mie fluid characterized by the exponent pair (n_k, m_k), with $k = 1, \ldots, K$. Data set k contains N_k retained state points indexed by $l = 1, \ldots, N_k$. For state point l of fluid k, the entropy scaling input variable is denoted by $\tilde{s}_{\mathrm{conf},k,l}$, and the corresponding self-diffusion target value is denoted by $y^{(D)}_{k,l}$.

Only the parameters $\alpha_2^{(D)}$ and $\alpha_3^{(D)}$ were fitted. The self-diffusion parameter vector used in the hierarchical fit is therefore

$$\underline{\alpha}^{(D)}(n,m) = \left(\alpha_2^{(D)}(n,m), \alpha_3^{(D)}(n,m)\right). \tag{S2}$$

For a given data set k, the entropy scaling model prediction for state point l is written as

$$y_{k,l}^{(D),\text{model}} = f^{(D)}\left(\tilde{s}_{\text{conf},k,l}; \underline{\alpha}^{(D)}(n_k, m_k)\right), \tag{S3}$$

where $f^{(D)}$ denotes the form of the entropy scaling correlation given in Eq. (18) of the main manuscript and returns $\ln(\hat{D}^{\text{o}})$.

Polynomial representation of the exponent dependence

In the hierarchical formulation, each scalar entropy scaling parameter $\alpha_j^{(D)}$ is represented as a smooth function of the Mie exponents (n, m). A low-order polynomial basis centered at the Lennard-Jones reference exponents $(12, 6)$ was used:

$$\psi_r(n,m)_{r=1}^{N_\psi} = \left[1, n-12, m-6, (n-12)^2, (n-12)(m-6), (m-6)^2\right], \tag{S4}$$

with $N_\psi = 6$. The centered representation was chosen so that the first basis coefficient represents the value of the respective entropy scaling parameter at the Lennard-Jones reference point $(n, m) = (12, 6)$, while the remaining terms describe first- and second-order deviations from this reference.

Each scalar parameter is then written as

$$\alpha_j^{(D)}(n,m) = \sum_{r=1}^{N_\psi} \theta_{jr}^{(D)} \psi_r(n,m), \qquad j \in \mathcal{J}^{(D)}, \qquad \mathcal{J}^{(D)} = \{2, 3\}. \tag{S5}$$

Here, $\theta_{jr}^{(D)}$ are global regression coefficients. The coefficients are collected in the vector $\underline{\Theta}^{(D)}$, which denotes the complete set of global coefficients fitted for the self-diffusion coefficient. The fluid-specific parameter vector $\underline{\alpha}^{(D)}(n_k, m_k)$ is then obtained deterministically from Eq. S5.

Least-squares objective

The global coefficients $\underline{\Theta}^{(D)}$ were obtained by minimizing a sum-of-squares objective over all pure-fluid data sets and all retained state points:

$$\underline{\Theta}_{\text{opt}}^{(D)} = \arg\min_{\underline{\Theta}^{(D)}} \mathcal{S}\left(\underline{\Theta}^{(D)}\right), \tag{S6}$$

with

$$\mathcal{S}\left(\underline{\Theta}^{(D)}\right) = \sum_{k=1}^{K} \sum_{l=1}^{N_k} \left[y_{k,l}^{(D)} - f^{(D)}\left(\tilde{s}_{\text{conf},k,l}; \underline{\alpha}^{(D)}\left(n_k, m_k; \underline{\Theta}^{(D)}\right)\right)\right]^2. \tag{S7}$$

Here, $\underline{\alpha}^{(D)}(n_k, m_k; \underline{\Theta}^{(D)})$ indicates that the fluid-specific entropy scaling parameter vector is calculated from the global coefficient vector $\underline{\Theta}^{(D)}$ through Eq. S5.

All data points were assigned equal weight in Eq. S7. Consequently, fluids and state regions with more retained data points contribute more strongly to the optimized coefficients. This choice was retained because the objective was designed to prioritize the overall predictive performance across the full investigated state-space coverage. A possible consequence is that sparsely sampled exponent combinations have a smaller influence on the final global coefficients than densely sampled ones.

Use of EOS-derived scaling quantities.

For the hierarchical fit used in the executable ES+EOS framework, the target quantity $\hat{D}^{\text{o}}$ was constructed using thermodynamic quantities obtained from the equation of state. In particular, the configurational entropy $\tilde{s}_{\text{conf}}$ and the reduced second virial coefficient B^* entering the *Chapman–Enskog* normalization were evaluated consistently with the EOS route used for prediction. This ensures that the fitted parameters are directly compatible with subsequent ES + EOS calculations.

For analyses that assess entropy scaling independently of the EOS, the corresponding scaling quantities can instead be obtained directly from the molecular-simulation route. In the main predictive framework, however, the EOS-consistent definition is used so that the same thermodynamic input route is employed during parameter fitting and model evaluation.

Parameter table.

The optimized coefficients $\theta_{jr}^{(D)}$ define the complete empirical Mie fluid entropy scaling

parameter model. They are given in the following table.

Table S3: Optimized coefficients $\theta_{jr}^{(D)}$ of the hierarchical empirical model for the Mie fluid entropy scaling parameters for the self-diffusion coefficient. The basis functions are defined in Eq. S4.

r	$\theta_{2,r}$	$\theta_{3,r}$
1	0.14405648	-0.985229
2	0.00545511	0.00771183
3	0.19070574	-0.05311182
4	0.00048588	-0.00016753
5	0.03692268	-0.00709605
6	-0.08365237	0.02416881

Infinite Dilution Models

The entropy scaling model parameters for the self-diffusion coefficients in infinite dilution for the Mie fluids in mixtures A and B are given in Table S4.

Table S4: Parameters for the entropy scaling model (cf. Eq. 18 in the main body of the text) for the diffusion in infinite dilution.

	α_2	α_3
$\ln\left[\hat{D}^{\infty}_{\mathrm{Mie}(12,5)}\right]$	-0.08143	-1.07916
$\ln\left[\hat{D}^{\infty}_{\mathrm{Mie}(16,6)}\right]$	1.55708	-1.35155
$\ln\left[\hat{D}^{\infty}_{\mathrm{Mie}(14,5)}\right]$	-0.95873	-0.53960
$\ln\left[\hat{D}^{\infty}_{\mathrm{Mie}(20,7)}\right]$	-1.85032	-0.57436

GE Simulations

For the mixed-exponent Mie fluids Mie $(8, 4)$, Mie $(20, 4)$, and Mie $(20, 8)$, the critical properties (cf. Table 1 in the main part of this work) were determined from vapor–liquid equilibrium (VLE) data obtained by molecular simulation. The VLE simulations were carried out with the grand equilibrium (GE) method[S1] using the ms2 simulation code.[S2,S3] Initial estimates for the coexistence pressure and the saturated liquid and vapor densities were obtained from the SAFT-VR Mie equation of state.[S4] These estimates were subsequently refined by three consecutive GE–NpT cycles. In each cycle, a GE simulation was used to determine the vapor-phase coexistence state from the liquid-phase input data. The resulting vapor pressure was then used in an NpT simulation of the liquid phase at the same temperature, from which updated liquid-phase properties were obtained for the next GE cycle.

All simulations were performed in reduced units. In the GE simulations, the Monte Carlo GE ensemble was used with a target acceptance ratio of 0.5. Each GE run comprised 10 000 initial NVT steps, 25 000 μVT steps, and 200 000 production steps. Results and statistical errors were sampled every 1000 and 5000 steps, respectively. The vapor phase was initialized with 1372 particles at an initial reduced density of $\rho^* = 0.001$. The intermolecular potential was truncated at $r_\mathrm{c} = 5\sigma$ using a center-of-mass cutoff scheme. The corresponding liquid-phase simulations were carried out in the NpT ensemble by molecular dynamics using a Gear predictor–corrector integrator with a reduced time step of $\Delta\tau^* = 0.001$. Each NpT simulation contained $N = 4096$ particles and consisted of 20 000 initial NVT steps, 280 000 NpT equilibration steps, and 1 250 000 production steps. The pressure was controlled with Andersen's barostat using a piston mass of 10^{-4}. The chemical potential required for the GE calculations was determined by Widom test-particle insertion using 4096 test particles.

The critical temperature was obtained from the coexistence-density difference according to

$$\rho_\mathrm{L}^* - \rho_\mathrm{V}^* = a_\rho \left(T_\mathrm{c}^* - T^*\right)^\beta , \tag{S8}$$

where ρ_L^* and ρ_V^* are the saturated liquid and vapor densities, respectively, a_ρ is an adjustable coefficient, and β is the critical exponent. For Mie $(8,4)$ and Mie $(20,8)$, the critical exponent was fixed to the three-dimensional Ising value $\beta = 0.326$.[S5] For Mie $(20,4)$, $\beta = 0.35$ was used as an effective exponent to obtain a more stable extrapolation of the available coexistence data; this value is still close to the empirical one-third exponent commonly used for fluid coexistence curves.[S6] The parameters a_ρ and T_c^* were obtained by nonlinear least-squares fitting. The residuals were weighted by the statistical uncertainties of the saturated liquid and vapor densities. After fixing T_c^*, the critical density was determined from the law of rectilinear diameter,

$$\frac{1}{2}\left(\rho_\mathrm{L}^* + \rho_\mathrm{V}^*\right) = \rho_\mathrm{c}^* + b_\rho\left(T^* - T_\mathrm{c}^*\right), \tag{S9}$$

where ρ_c^* and b_ρ were fitted to the simulation data. The critical pressure was obtained by fitting the vapor pressure data to

$$p_\sigma^*(T^*) = \exp\left(a_p T^* + \frac{b_p}{T^*} + \frac{c_p}{(T^*)^4}\right), \tag{S10}$$

with the adjustable parameters a_p, b_p, and c_p. The critical pressure was then calculated as

$$p_\mathrm{c}^* = p_\sigma^*\left(T_\mathrm{c}^*\right). \tag{S11}$$

The critical properties reported in the main manuscript were obtained from Eqs. S8–S11.

Number of State Points Studied by MD

Tables S5 and S6 give the number of studied simulation data points per substance and mixture.

Table S5: Number of retained simulation data points after screening, per substance used in this study.

n	m	data points
8	4	332
8	6	325
10	6	324
12	4	325
12	5	326
12	6	312
12	7	277
12	8	253
14	6	280
16	6	272
20	4	320
20	6	248
20	8	206
36	6	223
48	6	212

Table S6: Number of retained simulation data points after screening, for the two mixtures.

Mixture	data points
A	115
B	120

Pure Component Results

This section provides additional results for the pure-component Mie fluids. The figures complement the discussion in the main text. Parity plots are shown to compare entropy scaling results obtained with EOS-derived thermodynamic quantities to those obtained directly from molecular-simulation data (cf. Fig. S1).

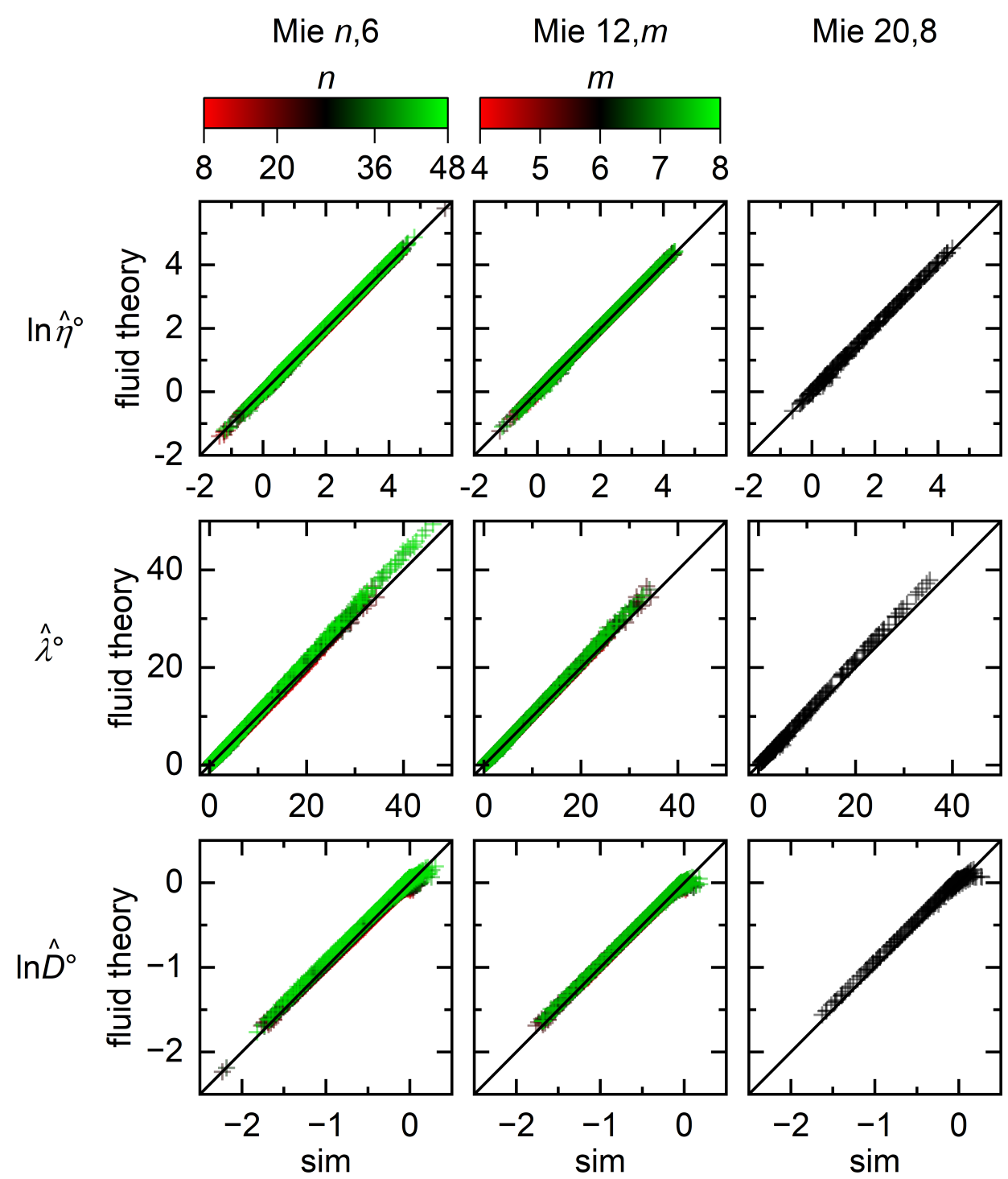

Figure S1: Parity plots comparing scaled transport coefficients obtained from the executable fluid-theory route with those obtained from the simulation-based entropy scaling route. The latter uses configurational entropies from molecular simulation and analytically evaluated virial coefficients.

Scaling Behavior of Different Fluid State Regions

Fig. S2 shows the assignment of state points to characteristic fluid regions for the 12,6 Mie fluid. Fig. S3 shows the entropy scaling behavior of the viscosity, thermal conductivity, and self-diffusion coefficient for these fluid regions. Overall, state points in the liquid, supercritical, critical, and high-density metastable regions exhibit good entropy-scaling behavior with low scattering. In contrast, an enhanced scatter is observed at low $\tilde{s}_{\mathrm{conf}}$, which corresponds mainly to gas-like states and low-density metastable states.

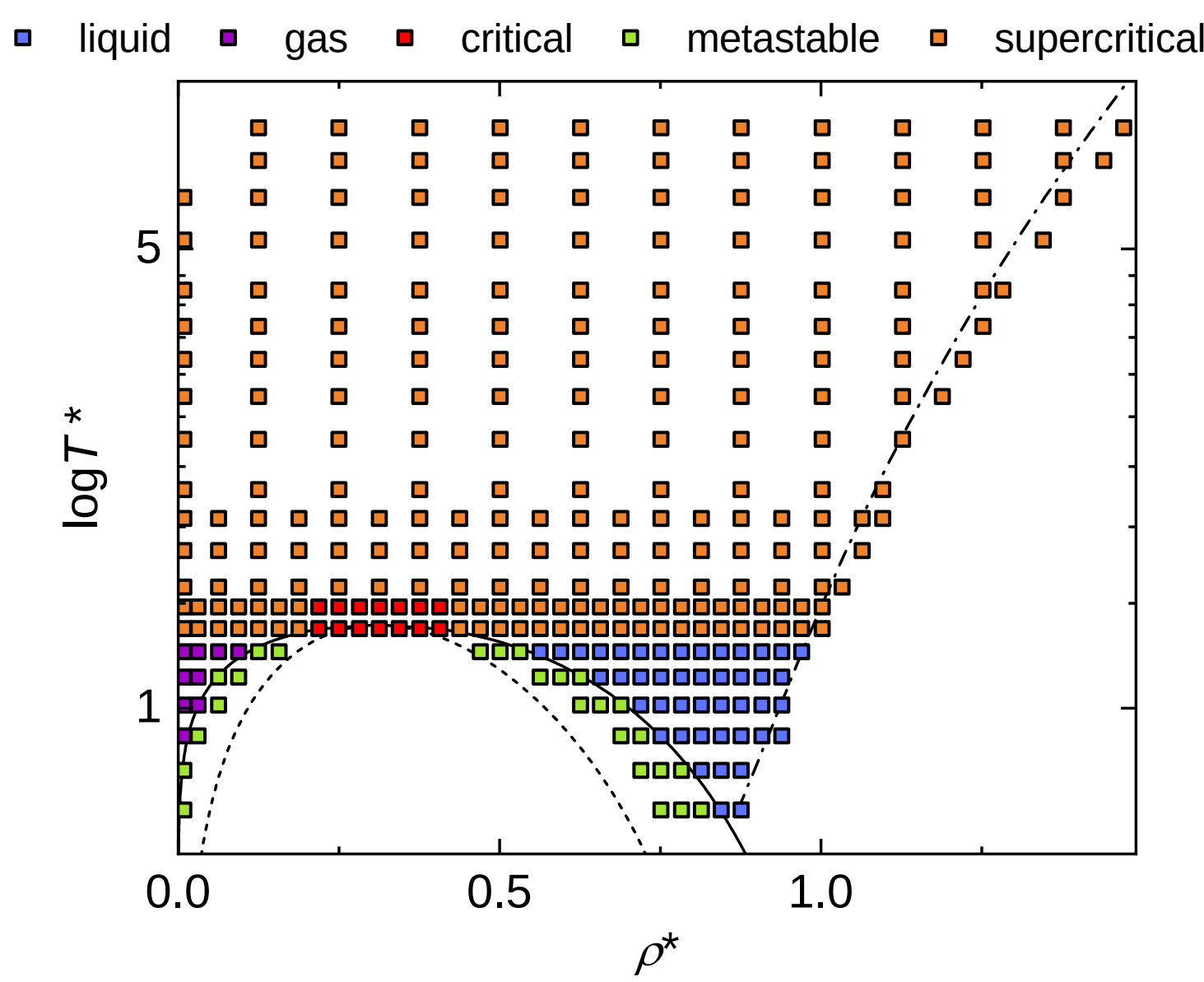

Figure S2: Simulation state points for the Mie $(12, 6)$ substance in reduced units with defined characteristic fluid regions. The critical region was defined as $0.98T_c < T < 1.1T_c$ and $0.65\rho_c < \rho < 1.38\rho_c$. The dash-dotted line shows the melting line from *Schultz and Kofke*.[S7] The binodal (solid line) and spinodal (dotted line) of the VLE were calculated by the equation of state from *Kolafa and Nezbeda*.[S8]

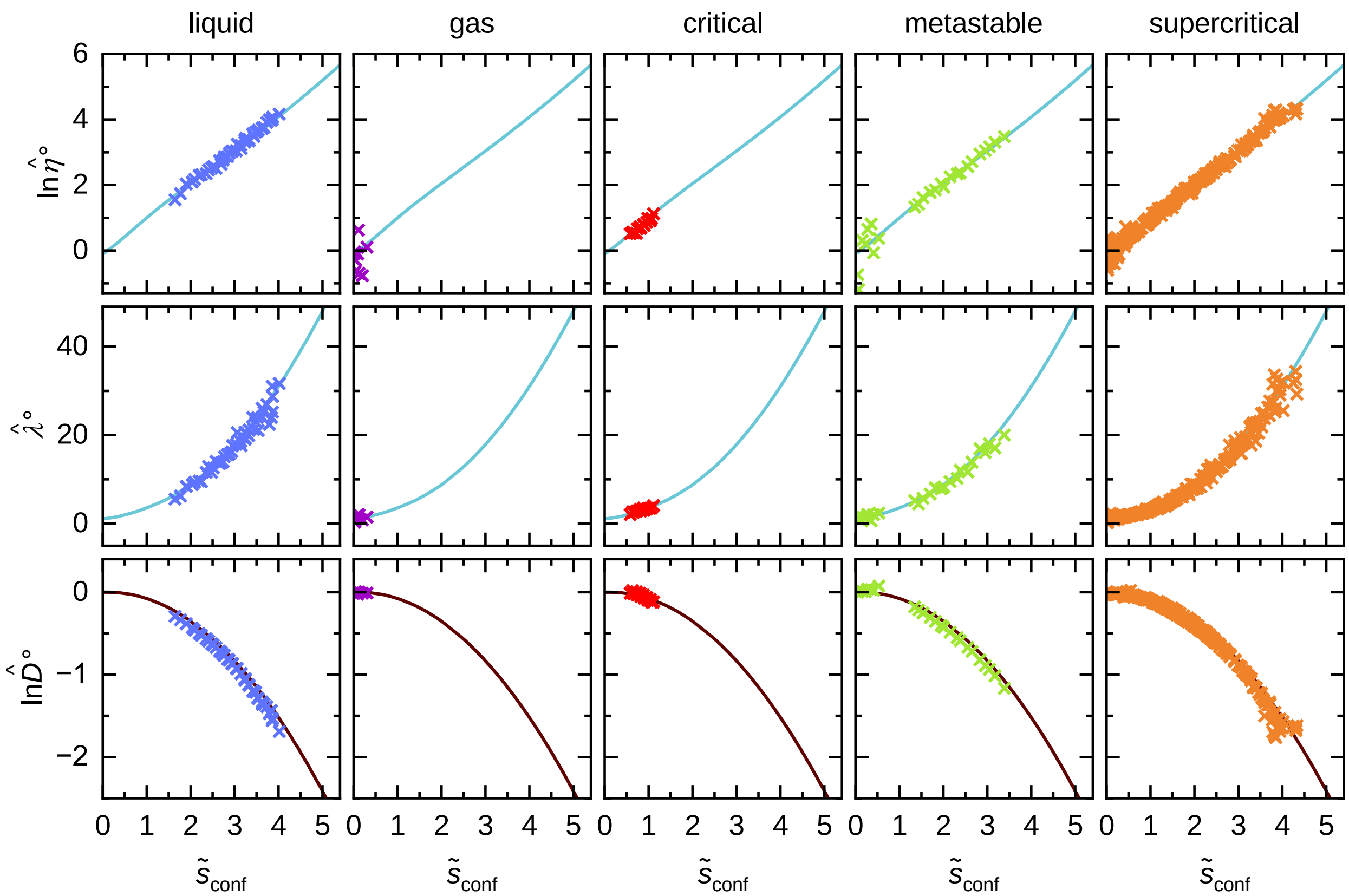

Figure S3: Entropy scaling behavior for the Mie $(12, 6)$ substance divided into the characteristic fluid regions shown in Fig. S2 for all three transport properties. Symbols depict the results based on configurational entropy from molecular simulations combined with analytically calculated virial coefficients. Solid lines represent the entropy-scaling model (cf. Eq. 18 in the main body of this work).

Binary Mie-Mixtures Results

This section provides additional mixture results for binary mixture A, consisting of Mie $(12, 5)$ and Mie $(16, 6)$, and binary mixture B, consisting of Mie $(14, 5)$ and Mie $(20, 7)$. The supplementary figures show the composition dependence of the viscosity, thermal conductivity, self-diffusion coefficients, and *Maxwell–Stefan* diffusion coefficients at different reduced temperatures and pressures (cf. Figs. S4–S9). The comparison of symbols and lines illustrates the agreement between molecular simulation data and fluid theory predictions across the full composition range. In addition, parity plots for the configurational entropy are provided to assess the consistency between the EOS-derived configurational entropies

and the corresponding values sampled in the MD simulations (cf. Fig. S10).

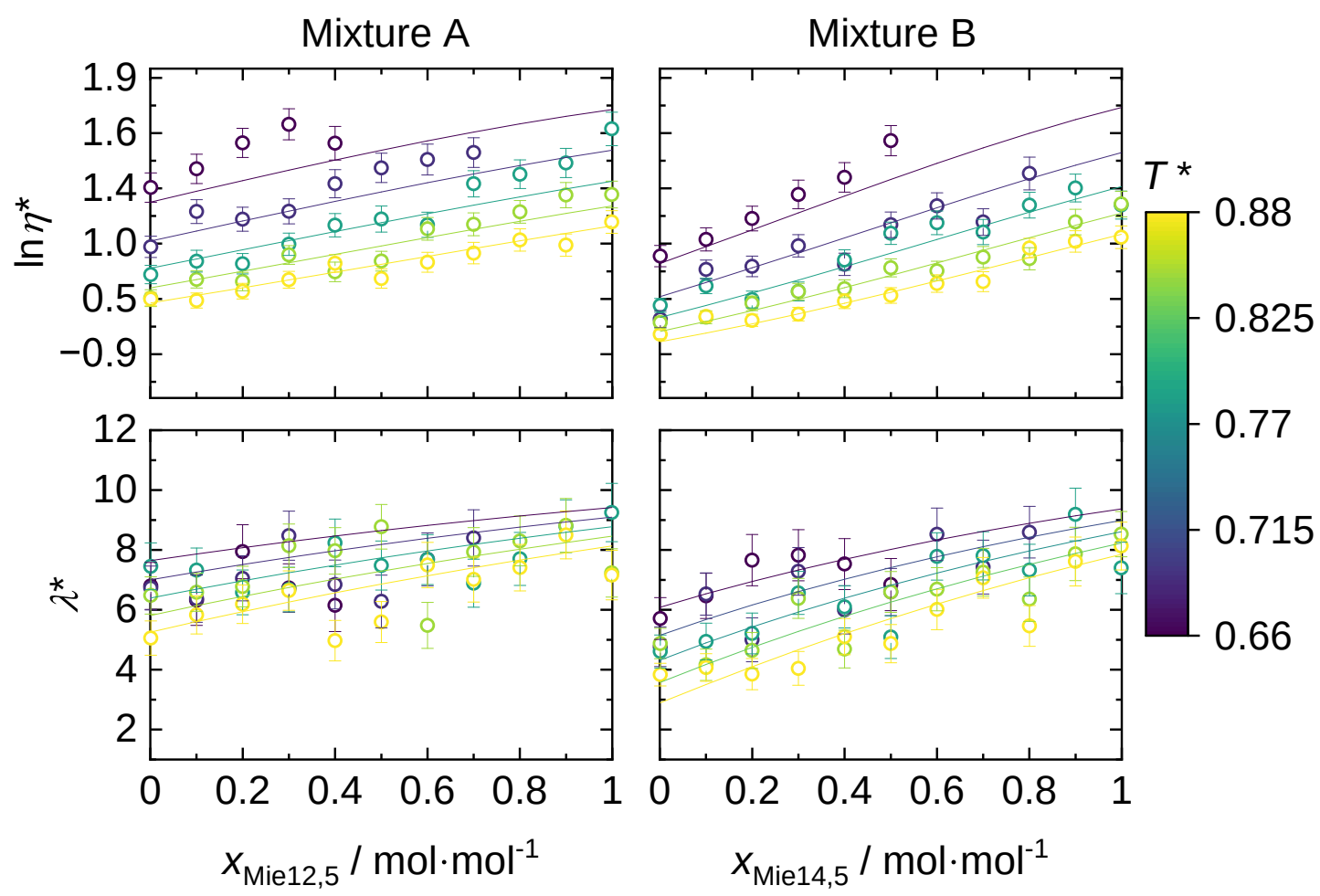

Figure S4: Viscosity and thermal conductivity for mixture A (Mie (12, 5) + Mie (16, 6)) and B (Mie (14, 5) + Mie (20, 7)) for $p^* = 0.2$. The color indicates the five different reduced temperatures $T^* \in \{0.66, 0.715, 0.77, 0.825, 0.88\}$. Symbols indicate simulation data, and lines represent predictions from the ES + EOS fluid-theory framework described in Eqs. 18 and 19 in the main body of the text.

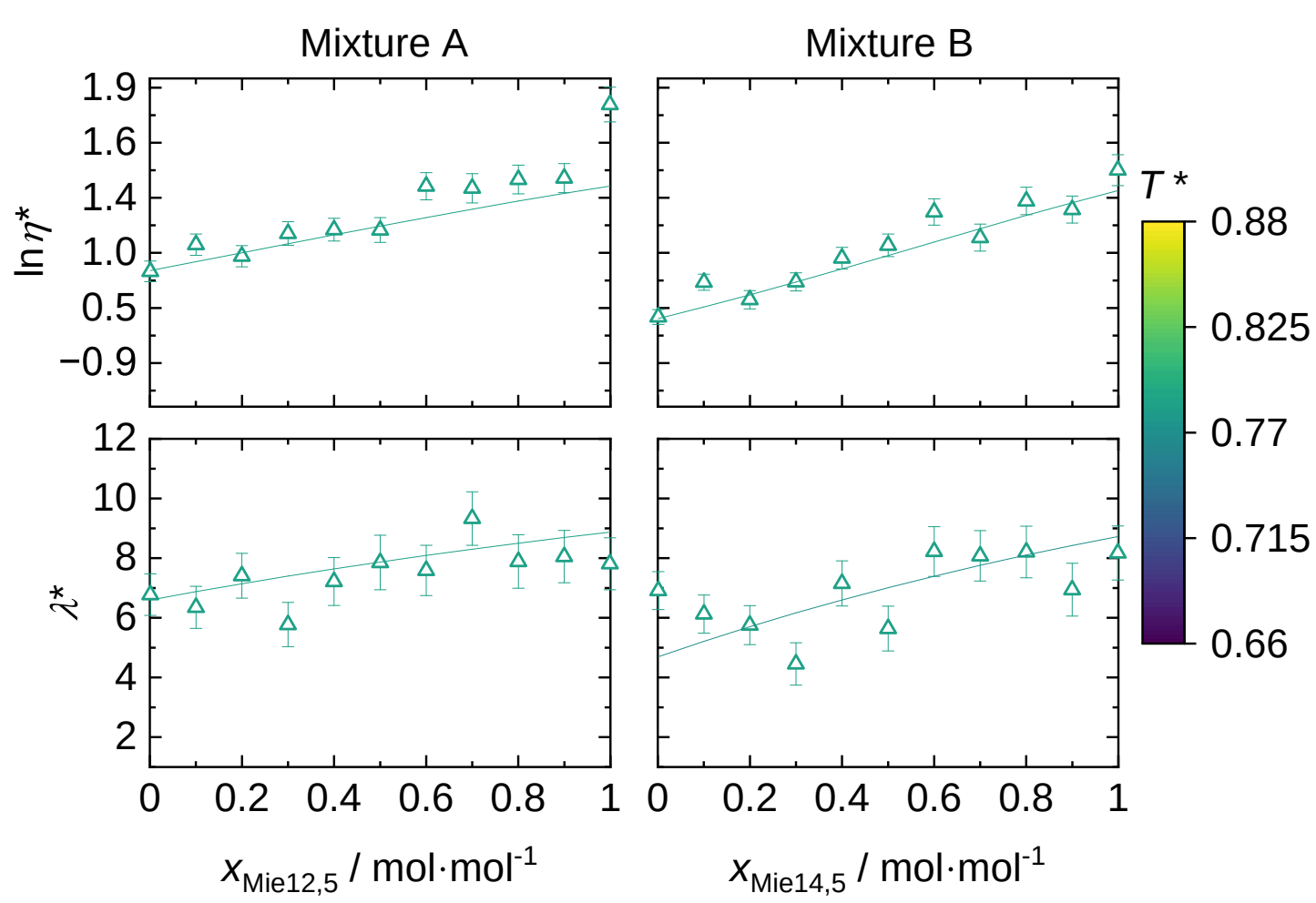

Figure S5: Viscosity and thermal conductivity for mixture A (Mie (12, 5) + Mie (16, 6)) and B (Mie (14, 5) + Mie (20, 7)) for $p^* = 0.3$. The color indicates the five different reduced temperatures $T^* \in \{0.66, 0.715, 0.77, 0.825, 0.88\}$. Symbols indicate simulation data, and lines represent predictions from the ES + EOS fluid-theory framework described in Eqs. 18 and 19 in the main body of the text.

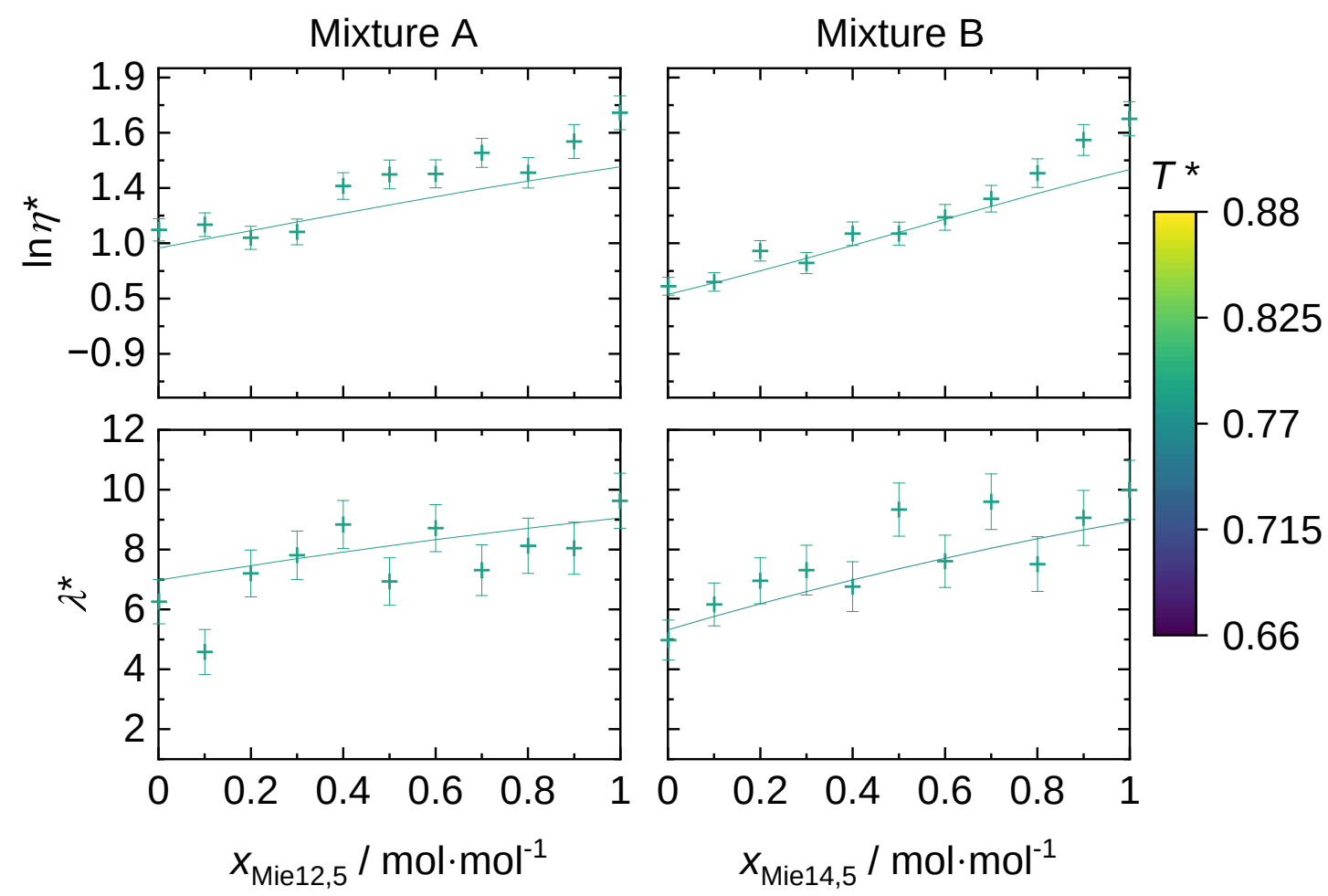

Figure S6: Viscosity and thermal conductivity for mixture A (Mie $(12, 5)$ + Mie $(16, 6)$) and B (Mie $(14, 5)$ + Mie $(20, 7)$) for $p^* = 0.5$. The color indicates the five different reduced temperatures $T^* \in \{0.66, 0.715, 0.77, 0.825, 0.88\}$. Symbols indicate simulation data, and lines represent predictions from the ES + EOS fluid-theory framework described in Eqs. 18 and 19 in the main body of the text.

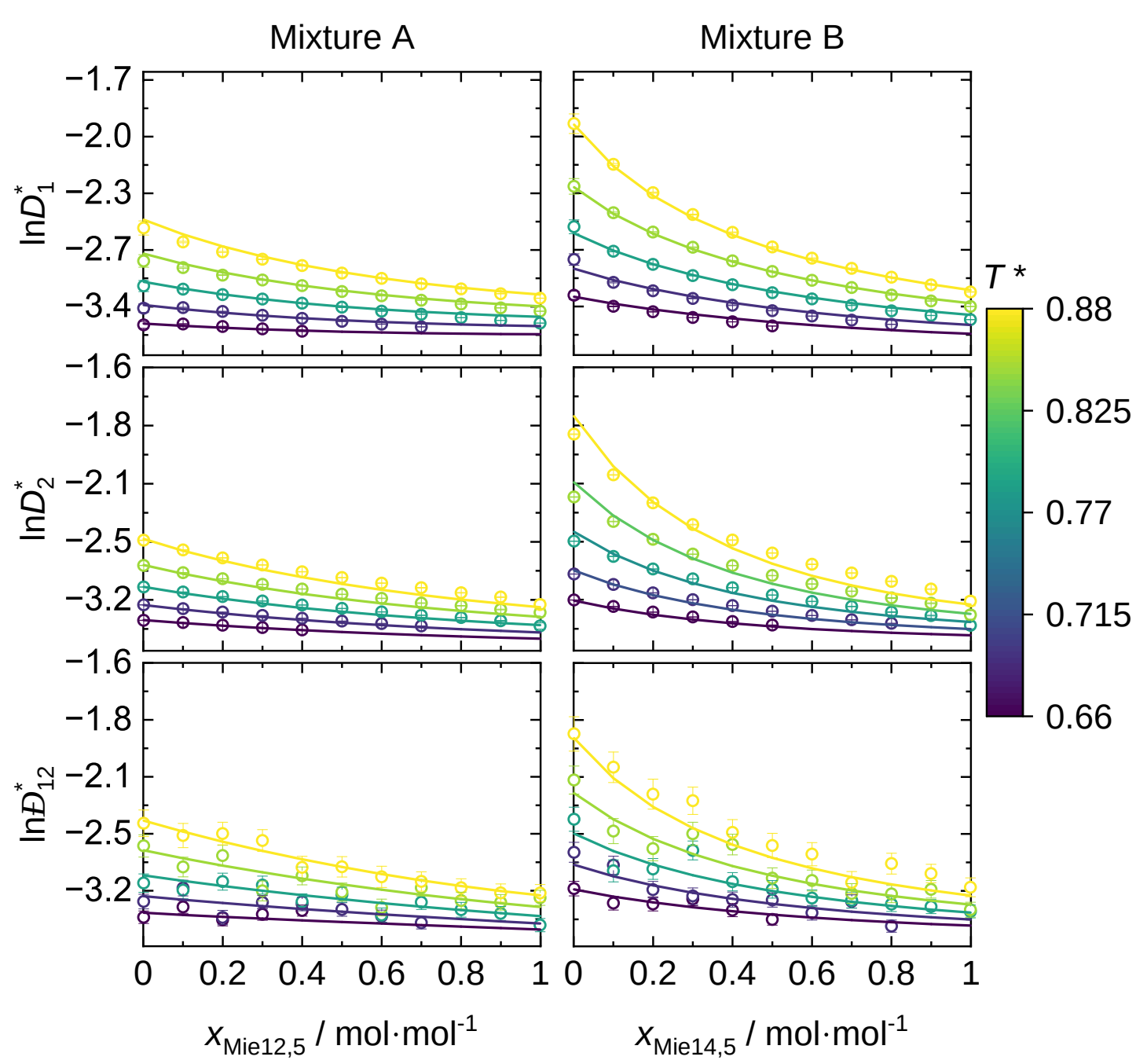

Figure S7: Diffusion coefficients for mixture A (Mie $(12, 5)$ + Mie $(16, 6)$) and B (Mie $(14, 5)$ + Mie $(20, 7)$) for $p^* = 0.2$. The color indicates the five different reduced temperatures $T^* \in \{0.66, 0.715, 0.77, 0.825, 0.88\}$. Symbols indicate simulation data, and lines represent predictions from the ES + EOS fluid-theory framework described in Eqs. 18 and 19 in the main body of the text.

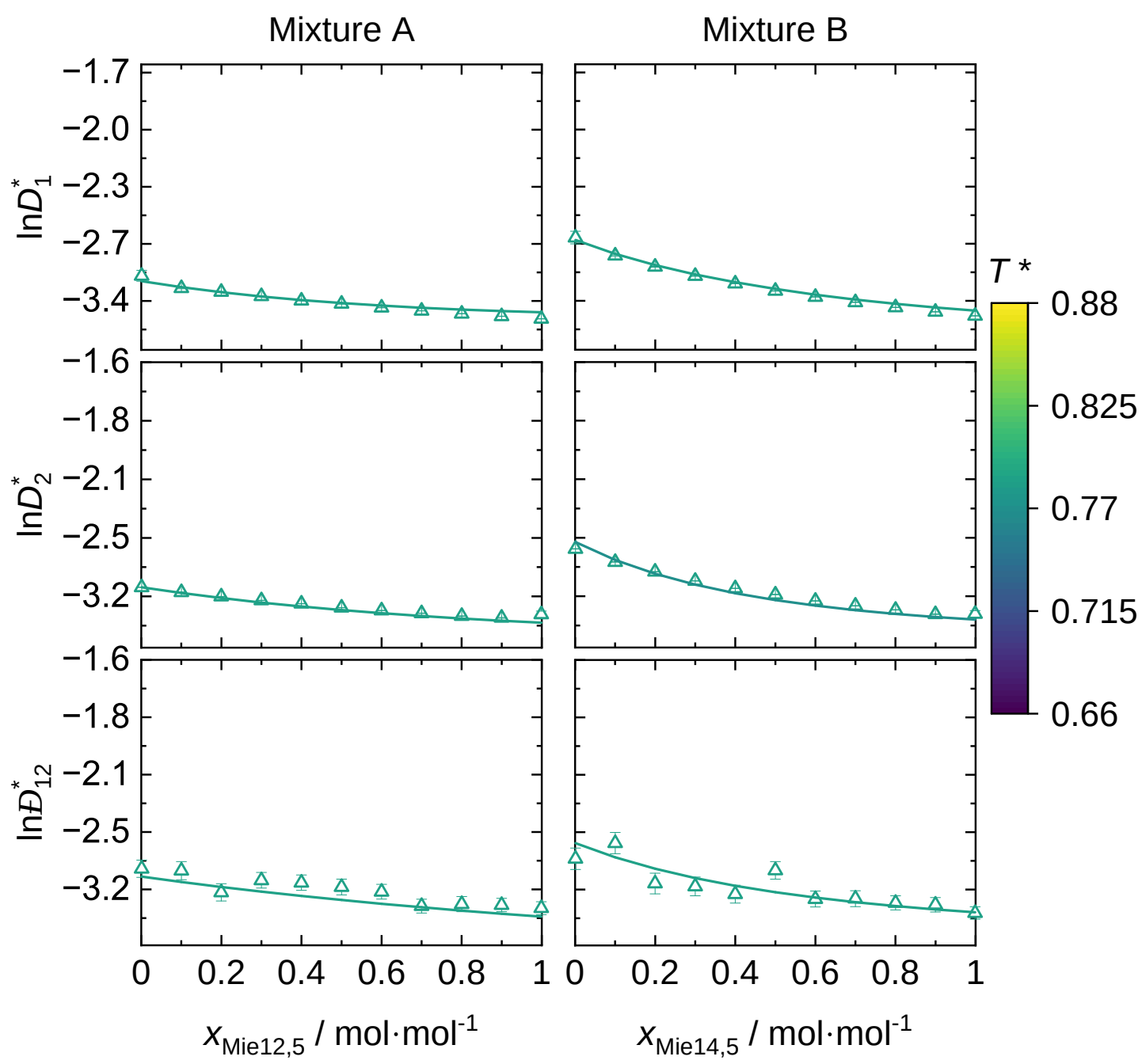

Figure S8: Diffusion coefficients for mixture A (Mie $(12, 5)$ + Mie $(16, 6)$) and B (Mie $(14, 5)$ + Mie $(20, 7)$) for $p^* = 0.3$. The color indicates the five different reduced temperatures $T^* \in \{0.66, 0.715, 0.77, 0.825, 0.88\}$. Symbols indicate simulation data, and lines represent predictions from the ES + EOS fluid-theory framework described in Eqs. 18 and 19 in the main body of the text.

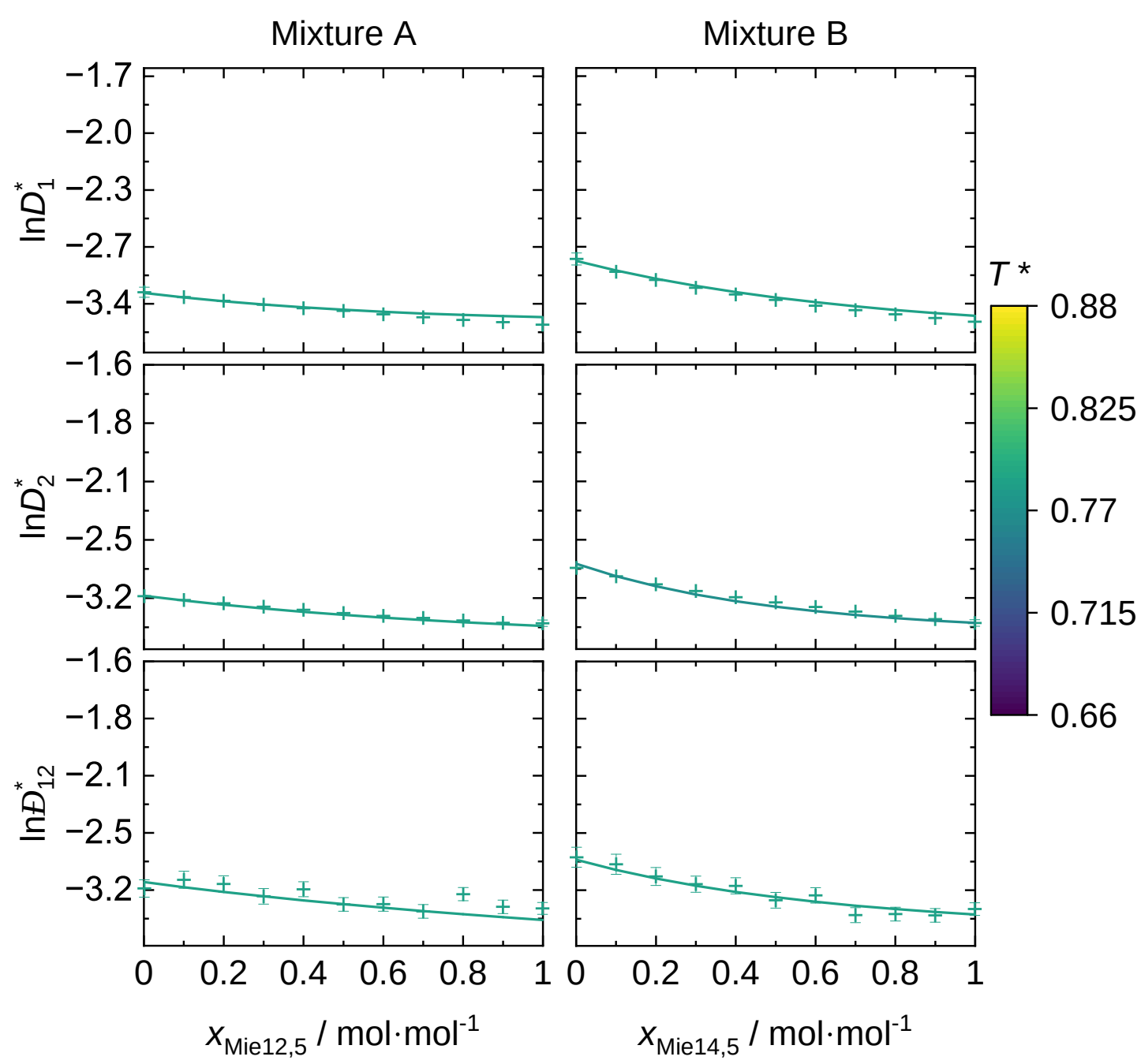

Figure S9: Diffusion coefficients for mixture A (Mie $(12, 5)$ + Mie $(16, 6)$) and B (Mie $(14, 5)$ + Mie $(20, 7)$) for $p^* = 0.5$. The color indicates the five different reduced temperatures $T^* \in \{0.66, 0.715, 0.77, 0.825, 0.88\}$. Symbols indicate simulation data, and lines represent predictions from the ES + EOS fluid-theory framework described in Eqs. 18 and 19 in the main body of the text.

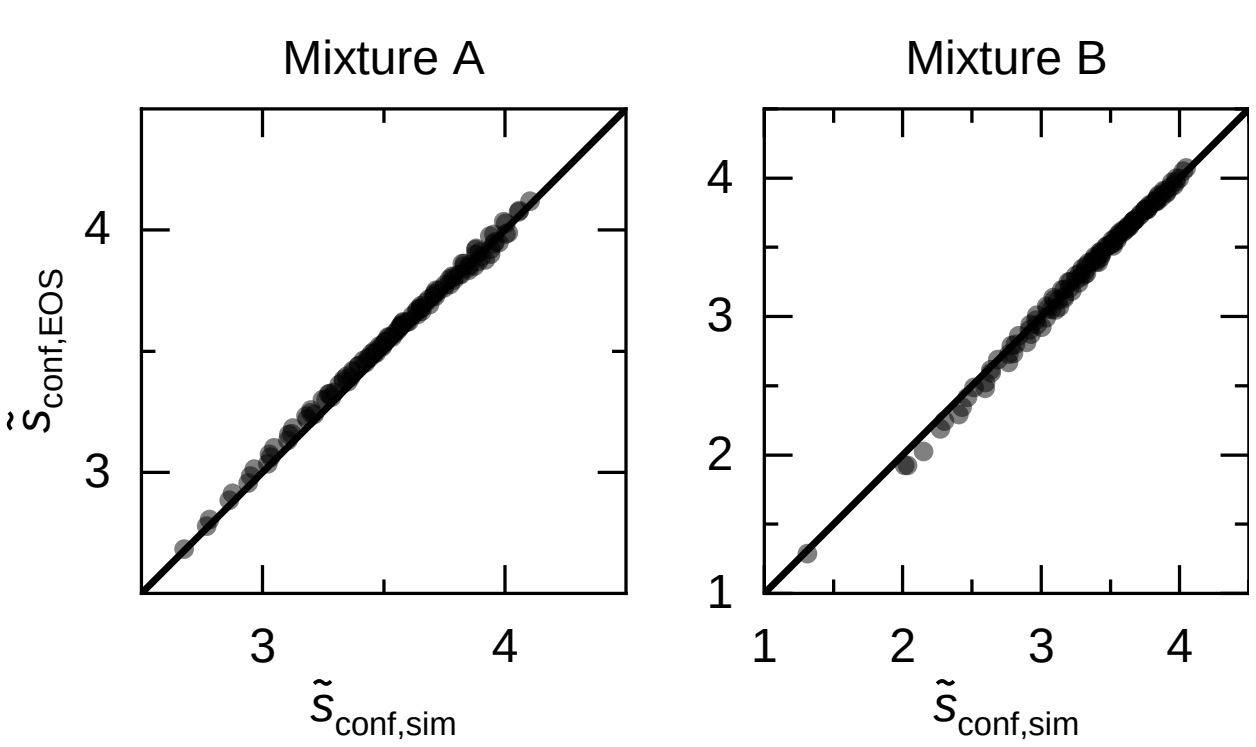

Figure S10: Parity plots for the configurational entropy from the EOS $\tilde{s}_{\mathrm{conf,EOS}}$ vs. simulation data $\tilde{s}_{\mathrm{conf,sim}}$ for the two binary mixtures A (Mie $(12, 5)$ + Mie $(16, 6)$) and B (Mie $(14, 5)$ + Mie $(20, 7)$).

Modeling Transport Properties of Mie Fluids

The available routes for modeling the transport properties of Mie fluids are based on different physical assumptions and, consequently, vary in terms of accuracy and range of applicability. Kinetic gas theory, in its revised *Enskog* form, commences with the collision dynamics between particles and requires no adjustable parameters. In the dilute gas it is rigorous and has recently been applied to Mie fluids for all four transport coefficients (including mutual diffusion) in Ref.[S9] However, as the density increases, the accuracy of this method deteriorates due to the breakdown of the two-body collision picture, which no longer sufficiently captures the correlated many-body motion present at higher densities. Thermodynamic (density) scaling is based on the approximate invariance of the reduced dynamics along lines of constant ρ^γ/T.[S10,S11] For inverse-power-law potentials, this scaling is exact which makes it particularly well suited to Mie $(n, 6)$ fluids. However, the scaling exponent γ is substance-specific, state-point dependent and must be determined from data. Entropy scaling replaces the two independent state variables with the configurational entropy, thereby directly coupling to an equation of state. This feature renders it attractive for practical applications. A comprehensive overview of the residual entropy scaling results proposed in recent years is provided in Ref.[S12] The price is a partly empirical formulation, particularly for the transition between dilute gas and dense fluid phases. The transition has been studied as a dynamic crossover between individual and collective contributions to viscosity.[S13] For mixtures, the need for additional assumptions, such as effective molecular mass and mixing rules for scaling parameters, has also been identified.[S14] Finally, data-driven correlations are both computationally cheap and reproduce training data well.[S15,S16] However, they lack thermodynamic consistency and provide limited physical insight. Additionally, their extrapolation behavior is not systematically regulated. The practical relevance of all these routes extends beyond model fluids, since Mie-type coarse-grained force fields are used for the simultaneous description of equilibrium, interfacial, and transport properties of real substances.[S17]

Literature Cited

(S1) Vrabec, J.; Hasse, H. Grand Equilibrium: Vapour-Liquid Equilibria by a New Molecular Simulation Method. *Molecular Physics* **2002**, *100*, 3375–3383.

(S2) Fingerhut, R.; Guevara-Carrion, G.; Nitzke, I.; Saric, D.; Marx, J.; Langenbach, K.; Prokopev, S.; Celný, D.; Bernreuther, M.; Stephan, S.; Kohns, M.; Hasse, H.; Vrabec, J. Ms2: A Molecular Simulation Tool for Thermodynamic Properties, Release 4.0. *Computer Physics Communications* **2021**, *262*, 107860.

(S3) Nitzke, I.; Guevara-Carrion, G.; Saric, D.; Homes, S.; Stephan, S.; Fingerhut, R.; Bernreuther, M.; Hasse, H.; Vrabec, J. Ms2: A Molecular Simulation Tool for Thermodynamic Properties, Release 5.0. *Computer Physics Communications* **2025**, *310*, 109541.

(S4) Lafitte, T.; Apostolakou, A.; Avendaño, C.; Galindo, A.; Adjiman, C. S.; Müller, E. A.; Jackson, G. Accurate Statistical Associating Fluid Theory for Chain Molecules Formed from Mie Segments. *The Journal of Chemical Physics* **2013**, *139*, 154504.

(S5) Pelissetto, A.; Vicari, E. Critical Phenomena and Renormalization-Group Theory. *Physics Reports* **2002**, *368*, 549–727.

(S6) Guggenheim, E. A. The Principle of Corresponding States. *The Journal of Chemical Physics* **1945**, *13*, 253–261.

(S7) Schultz, A. J.; Kofke, D. A. Comprehensive High-Precision High-Accuracy Equation of State and Coexistence Properties for Classical Lennard-Jones Crystals and Low-Temperature Fluid Phases. *The Journal of Chemical Physics* **2018**, *149*, 204508.

(S8) Kolafa, J.; Nezbeda, I. The Lennard-Jones Fluid: An Accurate Analytic and Theoretically-Based Equation of State. *Fluid Phase Equilibria* **1994**, *100*, 1–34.

(S9) Jervell, V. G.; Wilhelmsen, Ø. Revised Enskog Theory for Mie Fluids: Prediction of Diffusion Coefficients, Thermal Diffusion Coefficients, Viscosities, and Thermal Conductivities. *The Journal of Chemical Physics* **2023**, *158*, 224101.

(S10) Delage-Santacreu, S.; Galliero, G.; Hoang, H.; Bazile, J.-P.; Boned, C.; Fernandez, J. Thermodynamic Scaling of the Shear Viscosity of Mie n -6 Fluids and Their Binary Mixtures. *The Journal of Chemical Physics* **2015**, *142*, 174501.

(S11) Bell, I. H. Probing the Link between Residual Entropy and Viscosity of Molecular Fluids and Model Potentials. *Proceedings of the National Academy of Sciences* **2019**, *116*, 4070–4079.

(S12) Li, Z.; Yang, X.; Bell, I. H.; Duan, Y. Residual Entropy Scaling Model for Transport Properties of Real Fluids: A Review. *Industrial & Engineering Chemistry Research* **2026**, *65*, 13530–13557.

(S13) Bell, I. H.; Delage-Santacreu, S.; Hoang, H.; Galliero, G. Dynamic Crossover in Fluids: From Hard Spheres to Molecules. *The Journal of Physical Chemistry Letters* **2021**, *12*, 6411–6417.

(S14) Viet, T. Q. Q.; Khennache, S.; Galliero, G.; Alapati, S.; Nguyen, P. T.; Hoang, H. Mass Effect on Viscosity of Mixtures in Entropy Scaling Framework: Application to Lennard-Jones Mixtures. *Fluid Phase Equilibria* **2022**, *558*, 113459.

(S15) Šlepavičius, J.; Patti, A.; McDonagh, J. L.; Avendaño, C. Application of Machine-Learning Algorithms to Predict the Transport Properties of Mie Fluids. *The Journal of Chemical Physics* **2023**, *159*, 024127.

(S16) Chaparro, G.; Müller, E. A. Simulation and Data-Driven Modeling of the Transport Properties of the Mie Fluid. *The Journal of Physical Chemistry B* **2024**, *128*, 551–566.

(S17) Hoang, H.; Delage-Santacreu, S.; Galliero, G. Simultaneous Description of Equilibrium, Interfacial, and Transport Properties of Fluids Using a Mie Chain Coarse-Grained Force Field. *Industrial & Engineering Chemistry Research* **2017**, *56*, 9213–9226.
